# Movable surface acoustic wave tweezers: a versatile toolbox for micromanipulation

**DOI:** 10.1038/s41378-024-00777-3

**Published:** 2024-10-28

**Authors:** Xianming Qin, Xianglian Liu, Shuo Liu, Chuanyu Zhang, Ningning Bai, Xue Li, Weidong Wang, Dan Liu, Qiqi Yang, Ruiguo Yang, Yajing Shen, Xueyong Wei

**Affiliations:** 1 School of Mechano-Electronic Engineering, Xidian University, Xi’an, 710071 China; 2 State Key Laboratory for Manufacturing Systems Engineering, Xi’an Jiaotong University, Xi’an, 710049 China; 3 State Key Laboratory of Electromechanical Integrated Manufacturing of High-Performance Electronic Equipment, Xidian University, Xian, 710071 China; 4 State Key Laboratory of Holistic Integrative Management of Gastrointestinal Cancers and National Clinical Research Center for Digestive Diseases, Xijing Hospital of Digestive Diseases, Fourth Military Medical University, Xi’an, 710032 China; 5 Department of Biomedical Engineering, and Institute for Quantitative Health Science and Engineering (IQ), Michigan State University, East Lansing, MI 48824 USA; 6 Department of Electronic and Computer Engineering, The Hong Kong University of Science and Technology, Clear Water Bay, Kowloon, Hong Kong, China

**Keywords:** Electrical and electronic engineering, Microfluidics, Bionanoelectronics

## Abstract

Surface acoustic wave (SAW) tweezers are a promising multifunctional micromanipulation method that controls microscale targets via patterned acoustic fields. Owing to their device structure and bonding process, most SAW tweezers have limitations in terms of controlling the position and motion of the acoustic traps, as they generate an acoustic field with a fixed region and adjust the manipulation effects via signal modulation. To address this challenge, we propose movable SAW tweezers with a multilayer structure, achieving dynamic control of their wave field and acoustic trap positions; we demonstrate their precise manipulation functions, such as translation, in-plane rotation, out-of-plane rotation, and cluster formation, on a wide spectrum of samples, including particles, bubbles, droplets, cells, and microorganisms. Our method not only improves the degree of freedom and working range of SAW tweezers but also allows for precise and selective manipulation of microtargets via microtools and localized wavefields. Owing to their flexibility, versatility, and biocompatibility, the movable SAW tweezers can be a practical platform for achieving arbitrary manipulation of microscale targets and have the potential to play significant roles in biomedical microrobotics.

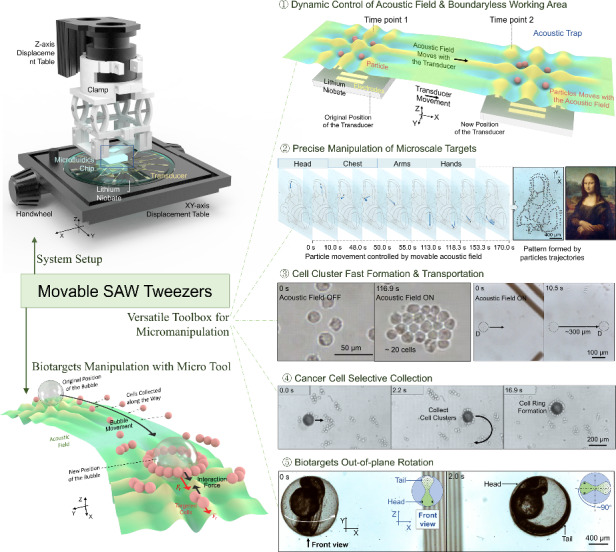

## Introduction

Surface acoustic wave (SAW) tweezers utilize interdigital transducers (IDTs) to generate acoustic waves to manipulate fluids and particles^1^ in a contact-free and biocompatible manner^2^. They can perform manipulation tasks, such as patterning^3^, sorting^4^, and mixing^5^, and are significant in biological, chemical, and medical fields^6^. To adapt to different requirements in various scenarios, SAW tweezers require a flexible acoustic field to achieve dynamic control of microtargets for different manipulation functions^7^. To this end, researchers have attempted various methods to realize tunable^8^, chirped^9^, or reconfigurable SAW tweezers^10^, such as with phase shifts^11^ and frequency modulation^12^. These methods can achieve rapid and accurate sorting^12^ or manipulation^13^ of particles^14^ or cells^13^ and rely on more complex device designs or signal inputs to expand functions or improve effectiveness; examples include tunable slanted-finger SAW tweezers^15^, which can dynamically adjust the location and spatial period of the acoustic field; wavenumber–spiral acoustic tweezers^16^, which can reshape wavefields to various pressure distributions; nanosecond-scale pulse SAW tweezers^17^, which can change the acoustic field region inside the polydimethylsiloxane (PDMS) channel; and bisymmetric coherent acoustic tweezers^18^, which can generate different complex wave-node arrays.

Unlike SAW tweezers, single beam or dual beam 3D acoustical tweezers^19–21^ can easily move their transducers to realize moveable and flexible acoustic fields and are suitable for applications requiring arbitrary patterning. Courtney et al. used an appropriate word to describe this advantage of 3D acoustical tweezers, i.e., dexterous^22^. In contrast, it is difficult for SAW tweezers to achieve dynamic translation and rotation of their acoustic field or switching and transformation between two different acoustic field types. The SAW field pattern follows the geometric structure of the transducer and can only change its position, direction, or wavelength within a small range. In addition, the transducer must be bonded with microchannels to transfer sound energy so that the action range and operation mode are fixed. The structures of the devices limit their flexibility and further limit their potential for application in micromanipulation instruments. Durrer et al. commented on this dilemma among acoustofluidic lab-on-a-chip devices when introducing their robot-assisted acoustofluidic end effector: despite their potential synergies, each has grown separately, and no suitable interface yet exists^23^.

In this work, we propose a novel SAW tweezer system with movable acoustic fields. This system has independently encapsulated channels and a multilayered structure to transfer acoustic waves from the piezoelectric substrate to the channel. The relative motion between the piezoelectric substrate and the channel allows for translation and rotation of the acoustic field, enabling the movable SAW tweezers to have a very large operation range, precise manipulation performance, and various functions, including translation, in-plane rotation, and out-of-plane rotation of the microtarget.

Compared with other contactless micromanipulation methods^24–29^, such as optical^24^ and magnetic^25^ methods, this movable SAW tweezer method has no requirements for magnetism^26^, charge^27^, or optical characteristics^28^ of the object, such as the refractive index^29^, and can perform label-free manipulation on the basis of the target’s sound speed, size, and density. This approach not only inherits the advantages of traditional standing field devices, allowing the patterning of targets with its potential wells in a harmless way, but also allows direct and free motion manipulation.

Compared with other traditional SAW tweezer devices, the movable SAW tweezer device is capable of various functions that no other SAW devices can fully accomplish; it allows precise control of particle motion with travel distances in millimeters and accuracy at the micron scale, continuous in-plane rotation of complex geometric targets with arbitrary points as rotation centers in the XY plane, and controllable out-of-plane rotation of biotargets with dimensions ranging from tens to over a thousand micrometers. Furthermore, the movable SAW tweezers can use micro air bubbles as a secondary sound source to alter the local SAW field and as a tool to manipulate biological samples indirectly. With the coupled field constructed from the scattered field and SAW field, dynamic control of the local acoustic pressure distribution via microbubble translation can be achieved, and precise and spatially selective collection of cancer cells can also be achieved. With this method, the acoustic pressure distribution can be locally reconfigured according to the acoustic characteristics and geometry of the tool, rather than following only the transducer design and input signal, and allows for precise and complex operation of individual targets with generated acoustic interaction forces and minimal influence on other unintended samples.

We believe that this SAW tweezer system, with its flexibility and versatility, has the potential to work as a suitable interface to realize complex micromanipulation functions and operations and as a practical platform to bridge the gap between SAW tweezer innovations and the biological/clinical benchtop.

## Results

**Fig. 1:**
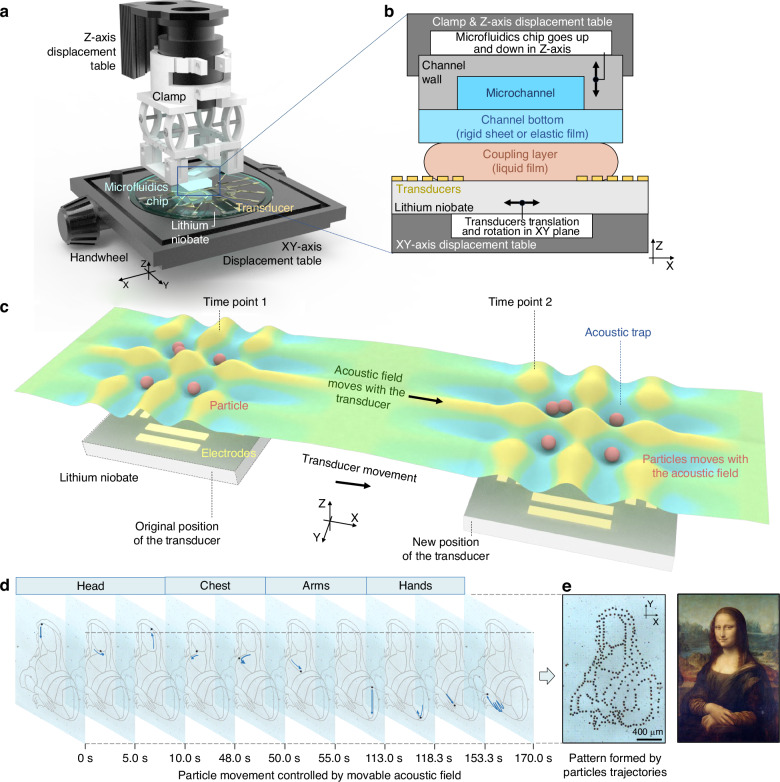
Experimental setup and working principle of the movable SAW tweezer system. **a** Movable SAW tweezer platform setup. **b** Multilayer structure of the device. **c**. Schematic diagram of the acoustic field movement method. **d** Control of the movement trajectory of a 31.1 μm particle by the movable acoustic field, with an input power of 9.8 W. The particle positions are captured frame by frame. **e** Mona Lisa painting and a Mona Lisa pattern depicted by the particle trajectories

The movable SAW tweezer system includes displacement tables, a customized clamp, and a SAW tweezer device (Fig. 1a). The SAW tweezer device has a multilayer structure (Fig. 1b): the piezoelectric substrate (lithium niobate) is fixed on the XY displacement table, whereas the independently packaged PDMS microchannel is fixed on the Z-displacement table through a 3D-printed clamp. The detailed structure of the clamp is provided in Supplementary Information Figure S1. A fluid coupling layer is used to connect the channel bottom and transducers, which allows the relative motion and transmission of acoustic waves.

The IDT generates acoustic waves based on input signal, which propagate along the surface of the piezoelectric substrate. The SAW leaks into the coupling layer fluid at the Rayleigh angle, passes through the coupling layer and the channel bottom, and forms a standing field inside the channel. The wave node pattern of the standing field in the XY plane follows the distribution of the SAW field on the piezoelectric substrate; therefore, with the movement of the transducer, the acoustic trap moves, which moves the particles (Fig. 1c). This intuitive method allows for direct control of particle motion (Fig. 1d) and precise operation of complex functions. In Fig. 1e, the dot draft of Mona Lisa is concatenated from frames of the trajectory of one 31.1 μm particle.

### Acoustic field in the multilayer structures

**Fig. 2:**
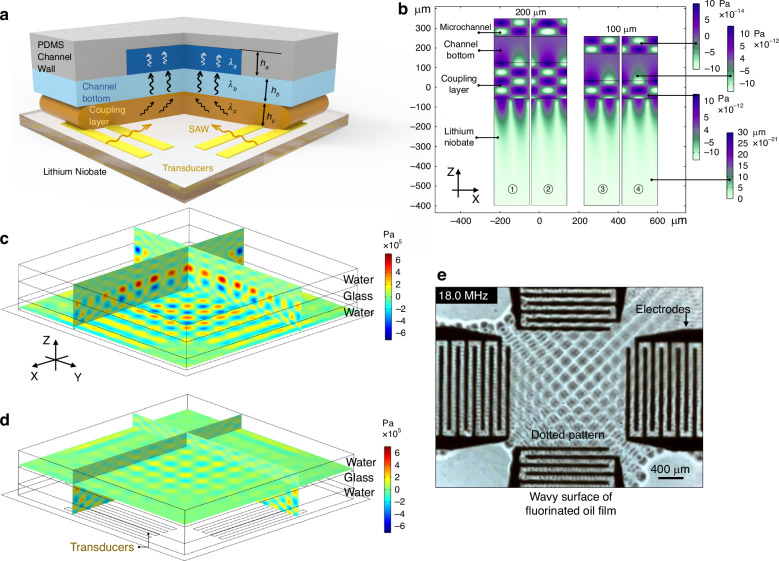
Acoustic wave transmission and pressure field distributions in the multiple layers. **a** Propagation of acoustic waves among layers of different materials. The mismatch in sound speed leads to wavelength variation. **b**. SAW modes and acoustic pressure COMSOL simulation results of the multilayer structure. The distributions of the 4 layers in every figure in **b** from top to bottom, are the acoustic pressure of the deionized water in the microchannel, the acoustic pressure of the glass sheet channel bottom, the acoustic pressure of the deionized water coupling layer, and the displacement of the lithium niobate piezoelectric substrate. The height of the coupling layer is 200 μm in ①&② and 100 μm in ③&④. The two figures in every pair (①&② or ③&④) are the two SAW modes, antisymmetric and symmetric. **c** The pressure field simulation result within the coupling layer and the changes in the spatial period of the acoustic pressure on the Z-axis. **d** The pressure simulation result within the microchannel. **e** The SAW pattern in the figure is the wavy surface of the fluorocarbon oil film, which is excited by leaky Rayleigh waves

In a multilayer SAW device^30^, an extra coupling layer^31^ can be used to conduct acoustic waves between the transducers and the fluid^32^. This kind of superstrate-based acoustofluidic device allows fluidic elements to be reversibly coupled to the transducer rather than bonded to it^33^, making the chip reusable and detachable. However, the acoustic impedance mismatch between the transducer and the propagation medium can lead to high signal reflections, and the sound speed mismatch among layers can alter the wavelength^34^. The acoustic wave propagates in the form of a SAW on the piezoelectric substrate, and the longitudinal wave propagates in the fluid coupling layer and continuous phase (Fig. 2a). In the middle of two orthogonal pairs of IDTs, the superposition of the orthogonal standing SAWs creates isolated wave nodes, whereas the wavelength variation causes the pressure field to have different spatial periods on the Z-axis (Fig. 2b–d). For a more intuitive display, a fluorinated oil film was added to the piezoelectric substrate, as shown in Fig. 2e to roughly depict the SAW field pattern. The wavelengths of the acoustic waves in the microchannel, channel bottom, and coupling layer and on the piezoelectric substrate surface are defined as ${\lambda }_{a}$, ${\lambda }_{b}$, ${\lambda }_{c}$, and ${\lambda }_{s}$, respectively, and the heights of each layer are ${h}_{a}$, ${h}_{b}$, ${h}_{c}$, and ${h}_{s}$, respectively (Fig. 2a). In addition, acoustic waves experience different types of acoustic attenuation in different materials, and their attenuation length is exponentially related to the speed of sound in the medium. For the interface between the piezoelectric substrate and fluid, the attenuation length on the substrate surface is proportional to the wavelength of the SAW^35^, whereas the attenuation length inside the fluid is proportional to the square of the wavelength of the longitudinal wave in the fluid^36^. Therefore, the key to designing multiple structures is to determine the materials and thicknesses of the channel bottom and coupling layer.

For the channel bottom, a PDMS film and borosilicate glass sheet are used in this study to encapsulate microchannels and conduct acoustic waves. Polymers are suitable for forming resonances in SAW devices, as their characteristic acoustic impedance is similar to that of fluids^7^. For layered resonators, to maximize the energy density in the fluid layer and achieve high acoustic attenuation, polymers can still be used as supporting layers, in which the losses can be acceptable as long as the system is well matched^7,37^. The characteristic acoustic impedance^38^ can be used to analyze the acoustic reflection and transmission. The characteristic acoustic impedance of the PDMS thin film is close to that of water, and it easily bonds with the PDMS channel wall. In contrast, the characteristic acoustic impedance of glass differs greatly from that of water, but the stiffness of glass makes it more robust and easier to use in experiments. For the channel bottom thickness, an excessively thick channel bottom exacerbates acoustic attenuation, whereas an excessively thin bottom layer increases bonding difficulty, reduces robustness, and is too fragile to be used in experiments. For example, for a “rigid superstrate–liquid coupler–LiNbO_3_” setup, a superstrate with a thickness between 0.35 ~ 0.7${\lambda }_{s}$ can yield a higher power density in the channel, regardless of the coupling layer material^33^. In this study, the thickness of the borosilicate glass sheet rigid superstrate is 130 ~ 160 μm, approximately 0.65 ~ 0.8${\lambda }_{s}$. The thickness of the PDMS film is 50 μm. Compared with the glass layer, the thin PDMS layer allows sound waves to experience less attenuation. However, the flexibility of the PDMS film makes it prone to bending when encapsulating large chambers, resulting in unstable manipulation performance in experiments. Therefore, 50 μm thick PDMS films are used to encapsulate narrower channels, with channel widths less than 1 mm, whereas glass sheets are used for large chambers, with widths greater than or equal to 1 mm.

For the coupling layer, a common practice in acoustofluidic devices is to add one or multiple layers of robust acoustic coupling materials to facilitate acoustic impedance matching and wave transmission. Efficient power transfer among layers can reduce Joule heating and mitigate potential piezoelectric crystal damage^39^. In this work, deionized water and the fluorocarbon oil FC-40 were used to connect the piezoelectric substrate and channel bottom. Compared with deionized water, FC40 has a lower sound speed (444 m/s) but a higher density (1.87 × 10^3^ kg/m^3^). Its characteristic acoustic impedance (8.30 × 10^5^ kg·s/m^2^) is closer to that of PDMS (1.04 × 10^6^ kg·s/m^2^) than to that of water (1.49 × 10^6^ kg·s/m^2^, at 25 °C), leading to a lower reflection coefficient ${R}_{p}$; however, a shorter wavelength may cause acoustic waves to experience greater attenuation at the same distance. In addition to materials, the thickness of the coupling layer affects the propagation of sound energy. We conducted a series of simulations for the vibration mode via simplified models with different coupling layer thicknesses. To ensure effective and stable acoustic manipulation, we expect high acoustic pressure and an acoustic pressure gradient in the microchannel, and the acoustic pressure distribution pattern in the microchannel is consistent with the surface acoustic wave distribution pattern on the piezoelectric substrate without distortion. As the coupling layer thickness increases, the distortion of the acoustic pressure distribution inside the microchannel increases (Fig. 2b). More simulation results are given in the supplementary file.

**Fig. 3:**
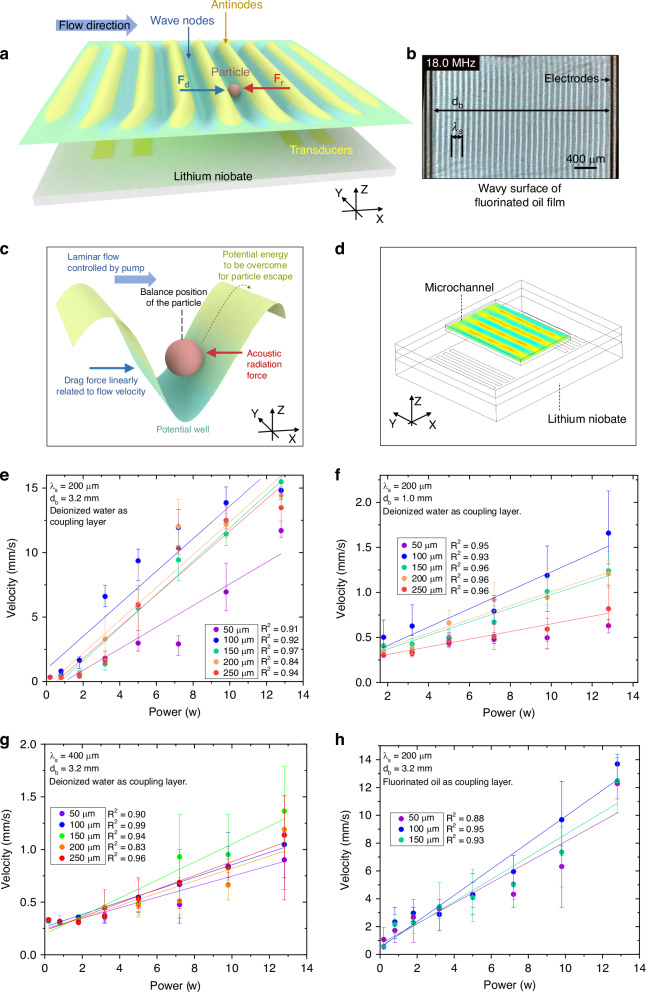
Influence of the coupling layer material and thickness on device performance. **a** Schematic diagram of the device performance testing experiments. **b** The corrugated surface of a fluorocarbon oil film is used to demonstrate the distribution of standing wave fields on piezoelectric substrates. The spatial period of the oil film surface on the XY plane is the same as the wavelength ${\lambda }_{s}$ of the SAW. **c** In a potential well, the particles maintain equilibrium under drag and gradient forces**. d** COMSOL simulation results of the pressure field in the microchannel. **e**–**h** The maximum flow velocities that allow the SAW tweezers to trap particles under specific input powers and coupling layer thicknesses. Each data point is the average of five measurements. **e**–**g** Show the performance of transducers with different wavelengths or distances ${d}_{b}$, whereas (**e**, **h**) show the performance of the same transducers but with different coupling layer materials

To analyze the effects of the coupling layer material and height on device performance, a series of tests were further conducted (Fig. 3). Two IDTs arranged in opposite directions are used to construct a linear-shaped standing wave field within the channel (Fig. 3a), so the particles can be trapped at the wave nodes. The flow velocity is perpendicular to the wave node, with its drag force ${F}_{d}$ collinear and opposite to the acoustic radiation force ${F}_{r}$ acting on the particles. In the Y direction, i.e., the length direction of the electrode finger (Fig. 3a), the linear standing wave field has a consistent distribution within the acoustic window area (Fig. 3b), whereas the dotted standing wave field has an interval distribution of wave nodes and antinodes (Fig. 2e). In the dotted field, with a high flow rate and high drag force, the particles may escape the acoustic trap by passing through the gap among antinodes. In contrast, the linear standing wave field can provide a unidirectional gradient force, so it is more suitable for the experiment to explore the relationship between the input power and flow velocity.

The experimental process was as follows. The acoustic field is turned on, and the particles are trapped while both the fluid and particles are stationary. At this point, the particles are located at the center of the wave node and are subjected to the gradient force from the antinodes on both sides (Fig. 3c). After that, the flow rate is gradually increased. The acoustic trap is a potential well surrounded by acoustic pressure (Fig. 3d). As the drag force increases, the particles slowly approach one side of the antinode and maintain equilibrium under the drag force and gradient force (Fig. 3c). When the flow rate reaches a certain threshold, the particles are pushed away by the drag force and moved for a distance no less than ${\lambda }_{s}$. We record the threshold flow rate as the maximum flow velocity ${v}_{m}$ that allows the tweezers to trap particles under a specific input power and coupling layer (Fig. 3e–h). For slowly moving micrometer-sized particles inside wave nodes, we can safely neglect their inertial effects^40^. The particle crossing an antinode along the X direction, i.e., the width direction of the electrode finger (Fig. 3a), means that the drag force on the particle is not less than the maximum value of the gradient force. After the particle passes the antinode, the gradient force and the drag force are in the same direction, pushing the particle toward the next wave node. The accelerated particle carries momentum, allowing it to pass through the next potential well with the same drag force.

The distance between two IDTs is defined as ${d}_{b}$. The particles used in this test are 31.1 μm in size. Transducers with various wavelengths ${\lambda }_{s}$ and distances ${d}_{b}$ have been tested in the experiments. The transducer with ${\lambda }_{s}$ of 200 μm and ${d}_{b}$ of 3.2 mm (Fig. 3e) have better performance when trapping particles than the ones shown in Fig. 3f, g. The fluorocarbon oil coupling layer does not differ from the deionized water coupling layer (Fig. 3e, h), and owing to the lipophilicity of the lithium niobate wafers, the oil film thickness has difficulty reaching a height of 200 μm. According to the experimental results, the maximum flow velocity ${v}_{m}$ increases with increasing input power in most cases.

In standing fields, microparticle movement is dominated by the acoustic radiation force and the drag force provided by laminar flow, while the drag force induced by acoustic streaming is generally much smaller than the acoustic radiation force^41^. When the compressibility, density, and speed of sound of the fluid; density, size, and speed of sound of the particles; and frequency of the SAW are fixed, the acoustic radiation force^42^ acting on a particle is determined by the acoustic energy density and position of the particle in the acoustic field^43^. Even within the acoustic trap, the acoustic pressure is not uniform, so sometimes, the particles are pushed a small distance by the drag force and stop again. In this situation, the particle does not break away from the acoustic trap but enters the position that provides the highest acoustic radiation force, which is why only the particles that move more than ${\lambda }_{s}$ are counted. The acoustic radiation force acting on particles is proportional to the input power and square of the applied voltage^40,44^, whereas the Stokes drag force is proportional to the relative velocity between the particles and the flow. Therefore, linear functions are used to fit the input power and velocity ${v}_{m}$. Figure 3c–f show that either too high or too low a coupling layer thickness reduces the acoustic energy density in the channel. In subsequent experiments, the thicknesses of the coupling layers were all 100 μm.

### Cancer cell translation, rotation, and aggregation

**Fig. 4:**
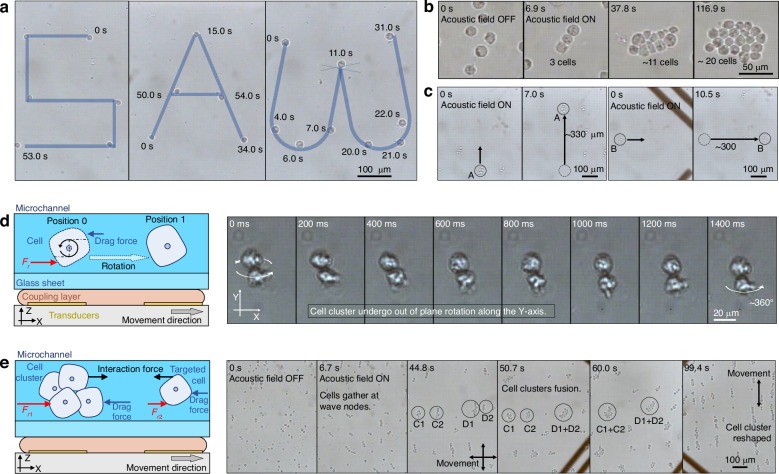
Manipulation of cancer cells. **a** Using the movement trajectory of cells to depict the word abbreviation “SAW”. **b** Guiding cells to form a cell cluster. **c** Cell cluster transportation. **d** Schematic and experimental diagrams of cell cluster rotation along the Y-axis. **e** Schematic and experimental diagrams of the fast formation method for cell clusters. In (**c**, **e**), different cell clusters are labeled with letters and numbers to prevent confusion

For biological targets such as cancer cells, microfluidic devices are regarded as safe and effective means of manipulation^45^. To further test the biocompatibility and performance of movable SAW tweezers on biotargets, we carried out a series of experiments on Caco-2 and THP-1 cells. The cells can be safely trapped inside the wave nodes and moved with the movable acoustic field (Fig. 4a). Because the propagation speed of acoustic waves in the multiple layers is much greater than the speed of the displacement table, the wave node pattern and the electrodes are relatively stationary during the movement of the transducer. For cells to move within the acoustic field, the acoustic radiation force should be no less than the viscous Stokes drag force generated by the motion of the cells relative to the continuous phase. Therefore, in a quiescent liquid, the upper limit of the movement speed of the transducer should monotonically increase with the input power, according to the results shown in Fig. 3. The cells used in the experiment shown in Fig. 4 were human colorectal adenocarcinoma cells (Caco-2). To achieve free manipulation of individual cells, we are able to assemble dispersed cells into cell clusters (Fig. 4b) and even achieve translation of cell clusters (Fig. 4c). However, regardless of the translation of cells or cell clusters, they are all in-plane motions. Furthermore, we achieved out-of-plane rotation of the cell clusters. As shown in Fig. 4d, moving the acoustic field along the X-axis can cause the cell cluster to rotate in the Y-axis direction. A controllable rotation function can help acquire comprehensive intercellular characteristics and obtain precise cellular positional identities in multidimensional optical imaging^46^.

For the acoustic field, a cell cluster is different in size and shape but has the same acoustic characteristics as an individual cell. On the one hand, considering that the acoustic radiation force has a cubic relationship with increasing target size, cell clusters do not require higher acoustic energy density for manipulation than individual cells do. On the other hand, in the acoustic field, cell clusters can provide acoustic interaction forces to attract nearby cells to construct larger cell clusters. This allows the acoustic field to form large cell clusters by moving cell clusters around to collect dispersed cells, which is easier than gathering cells one by one. By rapidly moving the acoustic field in the X or Y direction, that is, moving the acoustic field faster than the maximum velocity ${v}_{m}$, multiple cells can be trapped within the same wave node, pushed together into clusters due to the acoustic interaction force, and even reshaped by the acoustic field movement (Fig. 4e). The input power of the experiments in Fig. 4 is 9.8 W.

The method used in the above experiment provides a simple and intuitive means of manipulating cells. However, similar to many acoustofluidic micromanipulation methods, it still has several limitations. For example, acoustic waves affect all cells within the acoustic field region, applying a radiation force in the same way, making it difficult to independently manipulate individual cells. In addition, the acoustic field can only form cell clusters with nearby cells, which is conducted in a random and uncontrollable manner. The acoustic field can promote the formation of cell clusters, but it cannot determine which cells make up the cell clusters. This process lacks selectivity and accuracy. To further optimize the performance and functions of movable acoustic tweezers, we expect them to overcome these limitations and manipulate microscale targets in a manner similar to that of a microrobot. That is, instead of just holding or moving a target, the movable acoustic tweezers should hold and use a tool.

**Fig. 5:**
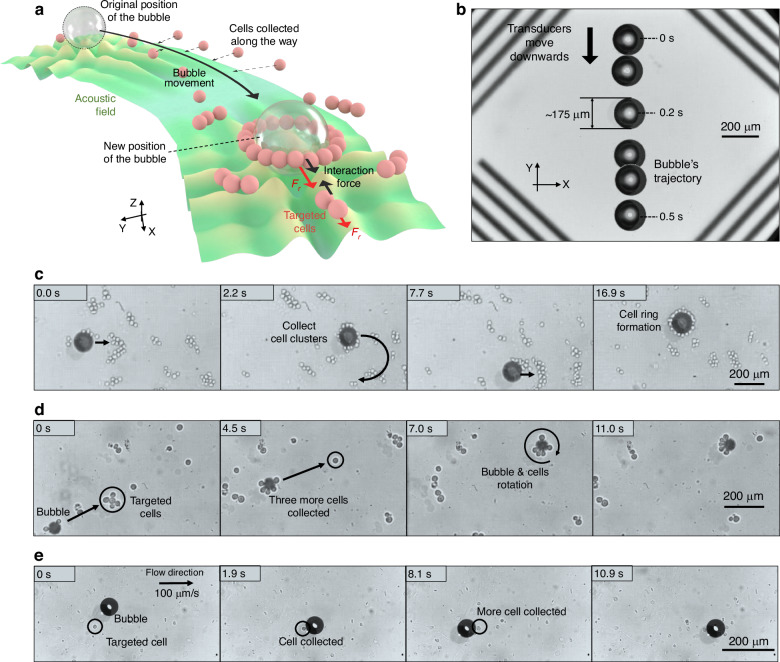
Utilizing air bubbles to collect cancer cells and form cell clusters. **a** Schematic diagram of the cell collection method. **b** Translation of air bubbles. **c** Utilizing bubbles to collect cancer cells and form circular cell clusters around bubbles. **d** Cell collection with smaller (60 μm) bubbles and rotation of the bubble‒cell system. **e** Specific cells can be collected with close placement of bubbles in a flow environment

However, since, as mentioned earlier, the acoustic field performs similar operations on all the targets inside the microchannel simultaneously, how do the tweezers distinguish between a tool and the manipulated target? We developed a simple method involving the use of a microobject that has a larger size or greater characteristic acoustic impedance difference (compared with continuous phases) than the manipulated targets, as a tool to control the targets indirectly. Through this method, the acoustic radiation forces on the tool and the target differ. That is, there will be a certain input power that allows manipulation of the microobject tool but allows the targets to stay put. In this way, we can use patterned acoustic fields to manipulate specific objects and then use these objects to finely and independently manipulate the target. In this study, we choose air bubbles as the microobjects and use them as tools to manipulate and collect cells (Fig. 5a).

As shown in Fig. 5, the air bubble can be translated by an acoustic field in the XY-plane (Fig. 5b) and can be further used as a tool to collect cancer cells (Fig. 5c). With different characteristic acoustic impedances, the bubble and cell experience different acoustic radiation forces, resulting in different resultant force directions under the influence of acoustic interaction forces. The air bubbles in Fig. 5b are approximately 175 μm in diameter and more than half a wavelength ${\lambda }_{s}$. The cells in the experiments shown in Fig. 5 are human leukemia monocytic THP-1 cells, which have been extensively used to study monocyte/macrophage functions, mechanisms, signaling pathways, and nutrient and drug transport. In this experiment, the primary acoustic radiation force drives the bubble in the XY-plane, whereas the acoustic interaction force from the bubble gathers the cells and forms a ring around the bubble rim (Fig. 5c).

In Fig. 5c, the bubble size is approximately 100 μm, which is close to the width of the wave node. The bubble in Fig. 5d is relatively small, approximately 60 μm in diameter. The size difference affects the number of cells that the bubble can collect. A smaller bubble, as shown in Fig. 5d, can capture fewer cells than larger bubbles can capture. In addition, when smaller bubbles are used for collection, the bubbles and the cells upon them may rotate in a certain direction. When a small bubble captures a small number of cells, the additional cells give this “bubble-cell” system a nonspherical structure, and the combined acoustic radiation force it receives generates a torque. In addition, when the size of the “bubble-cell” system is smaller than the wave node size, it will be able to rotate within the acoustic trap. In contrast, for larger bubbles, the collected cells do not significantly change the shape or size of the “bubble-cell” system, and the radiation force changes caused by additional cells are relatively small compared with the acoustic radiation force that the bubble originally experiences at its equilibrium position in the wave node. In addition, when the size of the “bubble-cell” system exceeds the size of the wave node, the gradient force from the antinodes blocks rotation. Figure 5c, d show how to collect cells in a stationary microfluidic environment. However, in practical biomedical applications, the scenarios can be more complex, e.g., capturing and extracting cells from flowing blood or other bodily fluids. Figure 5e shows the collection of moving cells in the flowing cell culture medium. The input power in the experiments shown in Fig. 5 is 7.2 W. The flow velocity of the cell culture medium in Fig. 5e is approximately 100 μm/s.

## Discussion

In this study, we propose a new kind of SAW tweezer system with a movable acoustic field, which can manipulate different types of microtargets and control their pattern and movement. This manipulation method, which is based on the relative movement of transducers and microchannels, is practical, direct, reliable, and highly versatile. We realized the precise manipulation of microtargets whose size ranged from 10 μm (particles) to over 1000 μm (zebrafish eggs), including particles, bubbles, microorganisms, cancer cells, and fish eggs, with operation functions including pattern, translation, rotation, deflection, and cluster formation.

The most basic feature of this movable acoustic tweezer system is the dynamic control of its acoustic field. One of the most common ways to control the acoustic field is signal modulation. Especially in the field of SAW tweezers, the signal modulation method is more popular than transducer displacement. One of the most important reasons for this is that the piezoelectric substrate needs to be bonded to the microchannels to conduct acoustic waves, leaving the transducers unable to move freely. To overcome this problem, independent encapsulated channels need to be constructed, and sound waves need to be conducted among different materials. For example, UV epoxy^30^, ultrasonic gel^31^, and deionized water^32^ can be used as coupling layer materials to transfer wave energy into microchannels. The channels can be encapsulated with glass, polydimethylsiloxane (PDMS), or polymethylmethacrylate (PMMA)^30–32^. Separating the microchannel from the transducer can avoid bonding problems. For example, the multifunctional acoustic tweezers proposed by Zhenhua Tian et al. can manipulate microtargets by attaching the transducer to Petri dishes and can allow the replacement of transducers to perform different functions^47^. These types of devices are usually considered detachable and reusable devices^48^, but with all these kinds of device formations, there is no SAW chip yet that can achieve dynamic motion control or microtool control. It would be very beneficial for practicalization and popularization if a contactless micromanipulation method could exert controllable yet calibratable force to move, hold, and guide micro/nanoobjects^24^ and assemble, pattern, reconfigure, and translate micro/nanoobject swarms^25^. For example, magnetic or electric microrobotics^26,27^, the optoelectronic method for microstructures fabrication^28,29^, and the bulk wave method for droplets transportation^49^. Compared with other contactless micromanipulation methods^24–29,49^, the standing SAW wave method can manipulate multiple targets continuously or simultaneously and form delicate patterns by trapping objects inside wave nodes, where the objects experience little acoustic radiation force, resulting in low damage to the operation and high biocompatibility. However, the operating range and degrees of freedom of the traditional SAW method are limited by the device structure, which makes it difficult to apply in micromanipulation platforms compared to other methods^19–22,24–29,49^. In this study, the system we propose shows the feasibility and versatility of a movable SAW field and, more importantly, breaks the chains of its own structure and gives full play to its advantages in a novel way. With further study in the future, this system is expected to be able to complete more functions and be utilized in more applications.

Stable acoustic wave transmission among layers is significant in SAW devices. Traditionally, multilayered superstrate-based SAW devices are considered disposable^44^ or detachable microfluidic devices^50^, and the coupling layer inside them is expected to avoid bonding and match the acoustic impedance. For a liquid coupling layer, the lack of thickness control can damage experimental performance and increase the difficulty in replicating results; structures such as spacers^51^ can help to stabilize the liquid film thickness but may introduce additional damping and serve as a source of error between theoretical analysis and experimental results^33^. The multilayer structure design process in this system is not limited to the connection between substrates but also includes the substrate leveling method and coupling layer thickness control, followed by material and geometrical parameter optimization of the multilayer structure to achieve dynamic control of a stable standing wave field with a high pressure gradient in the microchannel.

The advantages of movable SAW tweezers can be evaluated from the perspective of their manipulation function performance and can be described in detail in the following four aspects. The first aspect is the translation function. Compared with the directional propulsion of traditional SAW tweezers from traveling waves or potential well reconfiguration of the standing field, movable SAW tweezers can perform continuous and stable manipulation over a larger range (at the millimeter level and not limited by the acoustic aperture area) while maintaining high positioning accuracy and motion accuracy (Fig. 1 and Figure S7). The second aspect is the in-plane rotation function. For SAW tweezers with fixed transducers, rotation can only be performed inside the potential well and around the wave node point, and the rotation angle is not continuously variable. In this study, movable SAW tweezers can carry out continuous rotation on samples with several wavelengths and spiral structures around a random point several hundred micrometers away from the rotation center. The third aspect is the out-of-plane rotation function. By controlling the relative position between the antinode and the target, a movable SAW tweezer device can simply generate out-of-plane torque on microtargets and precisely control the rotation angle by the wavefield position and input signal power and duration, which few SAW devices can achieve. The fourth aspect is the spatially selective manipulation function. Movable SAW tweezers can utilize micro air bubbles to generate scattered fields and locally alter the background periodic pressure pattern. In this way, the bubble can act as a tool to precisely manipulate certain targets with minimal influence on other unintended targets. For each feature function, the movable SAW tweezer device has different operation methods than traditional SAW devices do and significantly improves manipulation performance. Furthermore, by integrating all the functions above into one device, movable SAW tweezers can switch their operation method freely, control the motion, gesture, and trajectory of the target at high degrees of freedom, and perform precise manipulation in a spatially selective manner.

The accuracy of the movable acoustic tweezer system is determined by multiple factors. At the center of the wave node, the acoustic radiation forces acting on the particle in all directions reach equilibrium. Therefore, the particle needs a small relative motion to the bottom of the potential well and enters the region with an acoustic pressure gradient to obtain the directional acoustic radiation force. However, the relative motion between the particle and the wave node only occurs during the translation process, and when the translation motion stops, the particle returns to the center of the wave node. Therefore, the trajectory of the particles follows the motion of the acoustic field and the transducer, with only a temporal delay. To achieve micrometer-level motion accuracy of particles, a displacement table with micrometer-level positioning accuracy is needed. Increasing the resonant frequency can reduce the size of the wave node and provide a higher acoustic radiation force for target manipulation. This is beneficial for improving the positioning accuracy of manipulation. However, it also reduces the acoustic attenuation length and makes it difficult for the acoustic wave to resonate in the channel after passing through the coupling layer and channel bottom. In addition, larger wave nodes and wave node lines allow the capture of large targets, such as 175 μm bubbles and microorganisms tens of micrometers wide and several millimeters long. Therefore, after comprehensive consideration, the design wavelength ${\lambda }_{s}$ of the key transducer with two orthogonal pairs of IDTs at the center of the wafer is set at 200 μm. By integrating transducers with different wavelengths, the wave node size can be changed in real time to adjust the control accuracy and the upper limit of the manipulated target size. The maximum operation region of the movable acoustic tweezer system is not limited by the acoustic aperture. Taking the two orthogonal pairs of IDTs as an example, although their acoustic field size is 1.4 mm × 1.4 mm, they can be moved to cover different areas. In fact, a smaller acoustic field size means more precise manipulation, which allows independent operations of adjacent targets, such as picking away unnecessary samples and leaving only the desired targets.

In addition to the direct manipulation of microtargets, we also demonstrate indirect manipulation with bubbles as microtools. The microtools with different characteristic acoustic impedances enable the acoustic field to manipulate individual targets precisely. In the experiment, we demonstrated a method of manipulating bubble tools with primary radiation forces and manipulating cells with an acoustic interaction force from the bubble tool. In fact, even without acoustic interactions, we can still rely on bubbles to push cells to specific positions, but the generated scattered field provides more possibilities for acoustic manipulation. In this study, we demonstrate the use of the movable SAW tweezer method to form cell clusters with cancer cells. The rapid formation of cell clusters (Fig. 4) and the selective collection of cell clusters (Fig. 5) are highly important in tissue engineering^52^, cell therapy, and drug development^53^. Acoustic assembly has the potential to overcome some of the challenges associated with cell cluster formation, especially for bottom–up tissue engineering, such as low density and lack of cell–matrix and cell–cell communication after formation^54^. Traditional SAW methods rely on the acoustic properties of cells for selective manipulation, and by introducing a movable acoustic field and bubble scattered field, we can improve the accuracy and controllability of the SAW cell assembly method. The method of using bubbles for acoustic manipulation is common in acoustofluidic devices at audible frequencies, inside which oscillating bubbles are mostly used to generate acoustic streaming and manipulate targets with drag force^55^. This process usually lacks selectivity. For movable SAW tweezers, through dynamic control of the microbubble and its scattered field, on-demand assembly of cells with identical sizes and acoustic characteristics was achieved.

In this method, we transform virtual tweezers into physical microscale tools. With further diversification of the tool structure in the future, this method is expected to achieve more complex functions. The translation and reconfiguration of the external field can be the basis of precise and dynamic control of microtargets^24–29,49^, and with more complex manipulation functions integrated, the boundary between virtual tweezers and microrobotics can be gradually blurred^28^.

## Conclusion

In summary, the SAW tweezer system provides feasible solutions for acoustic field movement and enables arbitrarily controlled motion of different targets and various types of functions, including precise translation, in-plane rotation, out-of-plane rotation, and cluster formation. This system can perform continuous trajectory control over long travel distances (at the millimeter level) while maintaining high positioning accuracy and motion accuracy, and it can perform stable operations on targets ranging from 10 μm (microparticles) to more than 1000 μm (fish eggs). Based on the movable SAW field, indirect and spatially selective manipulation with a coupled acoustic field is realized. The movable SAW tweezer device has the potential to perform as a suitable interface to link SAW tweezers with microrobotics and is expected to be a universal and dexterous platform to solve complex microtarget manipulation problems in the fields of biomedicine, applied chemistry, and health technology.

## Methods

### Device preparation

The piezoelectric substrate was a double-polished 128° Y-cut X-propagating lithium niobate (LiNbO_3_) substrate with a thickness of 1 mm. The transducers were fabricated via the lift-off process. The 30 nm Cr and 200 nm Au films of the IDT were deposited with an electron beam evaporator (TF500, Denton, UK). The LiNbO_3_ substrate with electrodes was cut into chip dies by an LED UV laser slicing machine (UVCS-15X, Hans Laser, CN). Chips were connected with wires through an electrode pad coated with conductive silver paste (LXZ919124, Luxianzi, CN). The silver paste was cured after heating at 85 °C for 6 h.

The PDMS microchannel was manufactured via soft lithography. An SU-8 photoresist (SU-8 GM1050, Gersteltec, Switzerland) patterned with a channel design was fabricated on a silicon wafer via photolithography. The base (Sylgard 184 Silicone Elastomer Base, Dow Corning) and the curing agent (Sylgard 184 Silicone Elastomer Curing Agent, Dow Corning) were mixed at a 10:1 volume ratio and placed under vacuum for degassing. The PDMS mixture poured on the silicon wafer formed solid polymer channels on the patterned SU-8 after heating at 80 °C for 4 h. A 50 μm PDMS film (KYQ 50 μm, Hangzhou Guinie New Materials Technology Co., Ltd., CN) and 100 μm borosilicate glass sheet (10212424c, Citotest Labware Manufacturing Co., Ltd., CN) were used as the channel bottom and connected with the PDMS channel via oxygen plasma bombardment. Deionized water and fluorocarbon oil FC-40 (FC-40, Shanghai Aladdin Biochemical Technology Co., Ltd., CN) were used as the coupling layer to connect the PDMS microchannel and piezoelectric substrate.

### Experimental setup

An AC signal generated by an arbitrary signal generator (AFG3022, Tektronix, USA) was amplified with a power amplifier (BA4850, NF, JP) and applied to the IDTs to generate the SAWs. The dynamic change in the experiment was recorded by a microscope (LV100, Nikon, JP) and a CCD camera (DS-Fi1, Nikon, JP). The flow rate was regulated by a pump (LSP01-2A, LongerPump, CN). Fluorocarbon oil (FC-40, biocompatible^56^, containing 5‰ surfactant) and deionized water were used as coupling layers in the experiments.

### Droplets and particles used in the experiments

The droplets used in this study were all deionized water, whereas the continuous phase was FC-40 fluorocarbon oil. The 10 μm particles (Rainbow, Tianjin BaseLine Chromtech Research Center, CN), 15 μm particles (Rainbow, Tianjin BaseLine Chromtech Research Center, CN), and 31.1 μm particles (Precision Size Polystyrene Microspheres, Suzhou Nanomicro Technology, CN) were polystyrene microspheres. Two kinds of particles were suspended in PBS (SS6235, Biofount, CN). A total of 1% sodium dodecyl sulfate (SS0013, Biofount, CN) was added to PBS to prevent particle aggregation.

### Biological samples

In the experiments, spirulina (Jiangsu Bluesea, CN) was injected into the microchannel through a pump. The fish eggs (Shandong YiXiYue Biotechnology, CN) were used within days post fertilization (dpf). Experiments on larvae until 5 dpf do not fall under animal welfare regulations. In this study, two types of cancer cells, Caco-2 and THP-1, were used to demonstrate the applicability of the device. Human colorectal adenocarcinoma cells (Caco-2 D611041-0001, Sangon Biotech (Shanghai), CN) were cultured in Dulbecco’s modified Eagle’s medium (DMEM-A12100S-500 ml, Shanghai Acmec Biochemical, CN), which contained fetal calf serum (M85271, Meryer (Shanghai) Chemical Technology, CN) and 1% penicillin/streptomycin (B645487-100 ml, Shanghai Boer Chemical Reagent, CN). The cells were harvested after they reached 80% confluence. Human leukemia monocytic cells (THP-1 D611021-0001, Sangon Biotech (Shanghai), CN) were cultured in RPMI 1640 cell culture medium (B645369-500 ml, Shanghai Boer Chemical Reagent, CN) containing 10% fetal calf serum (M85271, Meryer (Shanghai) Chemical Technology, CN) and 1% penicillin/streptomycin (B645487-100 ml, Shanghai Boer Chemical Reagent, CN). The cells were harvested when they were approximately 10^6^ to 10^7^ per milliliter.

### Simulation

The simulation was carried out with the commercial software COMSOL Multiphysics 6.0. In the three-dimensional COMSOL simulation, the size parameters and material settings of each structure were consistent with those of the actual experiments. The lithium niobate substrate was set as a piezoelectric material with mechanical damping. The crystal cut was applied by rotating the coordinate system on the basis of Euler angles. The loss factor defined in the frequency domain at the target frequency was converted to the Rayleigh stiffness damping parameter. The bottom surface and all the surfaces on the sides of the substrate were assigned low-reflecting boundary conditions. Since the effects of the IDT mass and stiffness on the dynamics of the device were not considered, the IDT was set as a 2D planar pattern rather than a 3D region and was defined as a perfect conductor. The model was solved for the target frequency defined by the electrode geometry. The finger width and interspace of the IDT were set to be the same. The acoustic window of the straight electrodes was 1000 μm, whereas the acoustic aperture of the arc-shaped electrodes of the focused IDT was 60°. The number of electrodes of each IDT was set to 4 for computational efficiency. For the four fingers of the IDT, two were grounded, two were applied with a MHz sinusoidal signal, and the voltage was set to 50 V. The simulation cannot calculate the coupling of the acoustic waves generated by all electrodes, so it can only roughly show the distribution of the acoustic pressure field. Corresponding to different experimental settings, the coupling layer can be set to be deionized water or fluorocarbon oil, while the channel substrate can be set to be glass or PDMS. The channel was set to be pure water at room temperature. The channel roof and the channel walls were set with an acoustic impedance of (1070 kg m^−3^ × 1030 m s^−1^) Pa s m^−1^, which reflects the PDMS boundary. The whole multilayer structure was composed of a solid piezoelectric substrate, a fluid coupling layer, a solid channel bottom, and a fluid microchannel. The interfaces among the piezoelectric substrate, coupling layer, channel bottom, and microchannel were all fluid‒structure interactions. The interface between the piezoelectric substrate and the coupling layer was set to be an acoustic-structure boundary. The two-dimensional COMSOL simulation was conducted via a similar method as the three-dimensional COMSOL simulation but with a simplified model, and the details of the two-dimensional COMSOL simulation are provided in the supplementary file.

## Supplementary information

Movie S1: Particle manipulation

Movie S2: Cancer cell manipulation

Movie S3: Indirect cancer cell manipulation

Movie S4: Selevtice cancer cell manipulation

Movie S5: Spirulina manipulation

Movie S6: Zebra fish egg manipulation

Supplementary file
