# Supplementary material for: Movable surface acoustic wave tweezers: a versatile toolbox for micromanipulation
Source: Microsyst Nanoeng. 2024 Oct 28;10:155. doi: 10.1038/s41378-024-00777-3 (PMC11519341; doi:10.1038/s41378-024-00777-3)
Supplement: Supplementary file 7 — Supplementary file [file 41378_2024_777_MOESM7_ESM.docx]

Supporting Information

Title

Movable Surface Acoustic Wave Tweezers: a Versatile Toolbox for Micromanipulation

Xianming Qin^1^, Xianglian Liu^2^, Shuo Liu^2^, Chuanyu Zhang^2^, Ningning Bai^1^, Xue Li^1^, Weidong Wang^1*^, Dan Liu^3^, Qiqi Yang^2^, Ruiguo Yang^4^, Yajing Shen^5^, Xueyong Wei^2*^

Affiliations

1. School of Mechano-Electronic Engineering, Xidian University, Xi’an 710071, China
2. State Key Laboratory for manufacturing Systems Engineering, Xi’an Jiaotong University, Xi’an 710049, China
3. State Key Laboratory of Holistic Integrative Management of Gastrointestinal Cancers and National Clinical Research Center for Digestive Diseases, Xijing Hospital of Digestive Diseases, Fourth Military Medical University, Xi’an 710032, China
4. Department of Biomedical Engineering, and Institute for Quantitative Health Science and Engineering (IQ), Michigan State University, East Lansing, MI 48824, USA
5. Department of Electronic and Computer Engineering, The Hong Kong University of Science and Technology, Clear Water Bay, Kowloon, Hong Kong, China

*Correspondence:

seanwei@mail.xjtu.edu.cn (X.W.)

wangwd@mail.xidian.edu.cn (W.W.)

Supplementary Notes

1. Movable acoustic tweezers and its manipulation method

In this system, from top to bottom are the Z-axis displacement table, customized clamp, microchannel device, ultrasonic transducer, and XY-axis displacement table. Instead of bonded or put on the piezoelectric substrate of the ultrasonic transducer like traditional SAW tweezers, the microchannel is connected to a detachable frame and lift up by the clamp (Figure. S1), so its channel bottom does not come into direct contact with the surface of the transducer, but with a coupling layer to conduct the acoustic waves. To optimize acoustic wave transmission, it is necessary to ensure that the channel bottom plane is parallel to the surface of the piezoelectric substrate. As shown in Figure. S1 **a** and **b**, the clamp is designed with four connecting shaft, and by squeezing the curved plate above the connecting shaft with screws, the height of the four connecting shafts can be adjusted separately. The black arrow in Figure. S1**c** represents the direction of the displacement boundary condition added at the screw hole. As shown in the Figure. S1**d**, the curved plate is squeezed along the X-axis by 200 μm on the screw hole position, resulting in a 60 μm displacement along the Z-axis on the bottom end of the connecting shaft, while the adjacent connecting shaft nearly stay still. The frame under the connecting shaft is detachable, which allows to replace different frames to fit different microfluidic devices.

The transducer is put under the microfluidic chip, generating acoustic waves into microchannel. The design wavelength of the 4-ports standing field transducer used in the experiment is 200 μm. As the acoustic field turned on, particles aggregated in the regions where the Gor'kov potential $U$ is minimum. As shown in Figure. S2, the wave nodes’ movement $\Delta d_{m}$ change the distribution of the Gor'kov potential, causing the particles originally in the acoustic trap exposed to unbalanced acoustic pressure. The acoustic radiation force $F_{r}$ acting on a particle with a diameter of $r_{p}$ can be described as followed ^[1]^:

$$\begin{aligned} F_{r}=-\nabla U=\nabla2\pi r_{p}^{3}\rho_{0}\left( \frac{\left\langle\boldsymbol{v}_{0}^{2} \right\rangle\left( \rho_{p}-\rho_{0} \right)}{\rho_{p}+\rho_{0}}+\left( \frac{\kappa_{p}}{\kappa_{0}}-1 \right)\frac{\left\langle p_{0}^{2} \right\rangle}{3\rho_{0}^{2}c_{0}^{2}} \right)\#\left( S1 \right) \end{aligned}$$

where the $\rho$, $c$ and $k$ are density, sound velocity and the compressibility, while the footnote “0” and “*p*” refer to the continuous phase fluid and the particle, respectively. $P_{0}$ is the pressure and $\boldsymbol{v}_{0}$ is the velocity in the fluid. With a $\Delta d_{m}$ on the wave node, the distances from the particle centroid to the surrounding antinodes also change. When only considering the X-direction movement $\Delta d_{m}$ of the transducer, the radiation force $F_{r}$ provided by the standing field can be simplified as followed ^[2]^:

$$\begin{aligned} F_{r}=\pi kr_{p}^{3}\kappa_{0}p_{0}^{2}\Phi sin\left( 2k\Delta d_{pw} \right)\#\left( S2 \right) \end{aligned}$$

where the acoustophoretic contrast factor $\Phi$ is a coefficient determined by the density and compressibility of liquids and particles, the wavenumber $k=2\pi/\lambda_{s}$, and $\Delta d_{pw}$ is the relative motion distance between the particle and the wave node. The acoustic radiation force $F_{r}$ and Stokes drag force $F_{d}=6\pi\eta{r_{p}v}_{r}$^[3]^ determines whether the particle can be moved by the motion of wave nodes. $v_{r}$ is the relative velocity of the particle in the continuous phase fluid, and $\eta$ is viscosity. In order for particles to follow the motion of the acoustic field, $F_{r}$ should be no less than $F_{d}$, so the transducer translation velocity $v_{tt}$ during particle manipulation experiment should be:

$$\begin{aligned} v_{tt}\leq\frac{r_{p}^{2}\kappa_{0}p_{0}^{2}\Phi k\sin\left( 2k\Delta d_{pw} \right)}{6\eta}\#\left( S3 \right) \end{aligned}$$

which changes with the particle position inside the wave node and the local acoustic pressure. At the position of the antinode, where the local acoustic pressure amplitude reaches maximum value $p_{0m}$, acquire the maximum transducer translation velocity $v_{mtt}$:

$$\begin{aligned} v_{mtt}=\frac{r_{p}^{2}\kappa_{0}p_{0m}^{2}\Phi}{6\eta}\#\left( S4 \right) \end{aligned}$$

In the simulation, the maximum value of acoustic pressure is given in the legend (Figure. S2). The $v_{mtt}$ and $v_{m}$ both reflect the device performance based on the balance of acoustic radiation force and drag force. $v_{mtt}$ is the maximum velocity of the transducer under ideal conditions. In practical tests, the device performance is evaluated with the measured maximum relative velocity $v_{m}$ between particle and fluid under certain input power.

Different from ideal situations, in actual experiment, particles are not volume free mass points and are influenced by acoustic pressure gradients from different regions and different direction. The acoustic gradient forces $F_{g}$ can be calculated according to ray acoustics, which indicates that acoustic waves can be approximated to a group of acoustic phonon rays carrying momentum $P$. The gradient force $F_{g}$ acting on the particle is ^[4]^:

$$\begin{aligned} F_{g}=\frac{nP}{c_{o}}\left( R\sin(2\theta_{i})-\frac{T^{2}\left( sin\left( 2\theta_{i}-2\theta_{r} \right)+Rsin\left( 2\theta_{i} \right) \right)}{1+R^{2}+2Rsin\left( 2\theta_{r} \right)} \right)\#\left( S5 \right) \end{aligned}$$

where $\theta_{i}$ and $\theta_{r}$ are the incident and refraction angles of acoustic phonon rays entering the target particle, respectively; $n$ is the refractive index of the surrounding medium; R and T are the Fresnel reflection and transmission coefficients, respectively. In the standing field, the radiation force’s scattering component in the direction of the incident wave has little effect on particle motion, while the gradient component in the direction of the intensity gradient play a leading role to trap and move particle.

As shown in Figure. S2, the asymmetry of acoustic pressure causes the particle to move towards one side. The “position 0” is the original position, when the transducer has not yet moved. The “position 1” is the new position when the transducer moved for a small distance $\Delta d_{m}$. The “position N” is the final position when the transducer moved for $d_{m}$, reach the destination and stopped, and the particle also moved for a certain distance. In Figure. S2, in order to distinguish the gradient force at different positions, the acoustic gradient forces $F_{g}$ in the standing field are marked with letters and numbers: “0” refers to position 0, and “1” refers to position 1. “l” and “r” refer to the gradient forces provided by the antinodes on particle’s “left” or “right”, respectively. “left” or “right” do not refer to certain actual direction or actual acoustic field region in experiment. They are only used to indicate the two acoustic pressure regions in Figure. S2**d**.

The “microchannel - channel bottom - coupling layer” structure causes the distribution of the pressure field can be influenced by various factors. The COMSOL simulation results of acoustic pressure field distribution in “water-glass-water” setup has been shown in Figure. 2. As a comparison and supplement, the COMSOL simulation results of acoustic pressure field distribution in “water-PDMS-oil”, “water-glass-oil” and “water-PDMS-water” setup are shown in Figure. S3. The “glass” refers to “borosilicate glass sheet”, “water” refers to “deionized water”, and “oil” refers to “fluorocarbon oil FC-40”. The acoustic reflection and transmission can be calculated as follow: When sound waves are incident vertically from the first medium to the second medium, whose characteristic acoustic impedances are $z_{1}$ and $z_{2}$, respectively. the pressure reflection coefficient $R_{p}$ ^[5]^is:

$$\begin{aligned} R_{p}=\frac{z_{2}-z_{1}}{z_{2}+z_{1}}\#\left( S6 \right) \end{aligned}$$

The characteristic acoustic impedance of borosilicate glass sheet is higher than that of deionized water. The pressure reflection coefficient $R_{p}$ between borosilicate glass sheet channel bottom and deionized water is also higher than that of “water-PDMS-oil” setup. As shown in Figure. 2 and Figure. S3, the systems with PDMS as channel bottom have higher acoustic pressure amplitude inside microchannel, compared to the systems with glass as channel bottom. That's why, although glass-bottom devices have better robustness and durability, and have been proven to be capable for particle manipulation, PDMS based devices are still retained as one of the options. Both deionized water and fluorocarbon oil can be used as coupling layer, however, the PDMS film without additional treatment is hydrophobic, therefore, fluorocarbon oil film and PDMS film can adhere better and have lower possibility of leaving bubbles between the channel bottom and piezoelectric substrate. In Figure. S3**g**, we further demonstrate the wave field at different frequencies with the wavy surface pattern of fluorocarbon oil film. As the same in Figure. 2**e**, the oil film is on the upper surface of the piezoelectric substrate, like a coupling layer. As shown in Figure. S3**g**, the wavy surface presents a clear dotted pattern at 18 MHz.

In Figure. S4, we used a two-dimensional simulation based on a simplified model to investigate the acoustic field among layers. For this simplified model, we chose a 200 μm wide area on XZ section as a basic unit, that is, the red rectangle in Figure. S4**a**. This XZ cross-section is located at the centerline of the acoustic aperture, and this basic unit is in the central region (center in X direction) of the electrodes. Most of the transducers used in this study are standing wave transducers with symmetrical ports, and tens of pairs of electrodes on each port. These electrodes are uniformly distributed in a spatial period along the X-axis, and no reflective gratings or delay lines on either side of the electrodes. Besides, for most transducers in this study, their acoustic aperture size is much larger than their wavelength, which weakens the influence of boundary effects on the acoustic field along the Y-axis. Therefore, this 2D area can be safely used as a periodic unit to simulate the vibration mode ^[6]^. For surface acoustic wave mode, the wave energy dies off within two to three wavelengths from the surface, so the height of the piezoelectric substrate in the unit don’t need to cover the whole thickness of the wafer. In this model, the piezoelectric substrate height is triple wavelength. This model is defined according to the 18 MHz transducer with a 200 μm wavelength, consist of the layers of lithium niobate piezoelectric substrate, deionized water coupling layer, borosilicate glass sheet channel bottom and the deionized water in microchannel. Interfaces including solid mechanics, electrostatics and pressure acoustics are used in this model. For structural boundary conditions, the piezoelectric material condition is set on the piezoelectric substrate domain section, and its lower boundary is set to be fixed. For electrical boundary conditions, the boundaries of the two terminals in the unit are set to be electrical ground and floating potential, respectively. For pressure acoustics boundary conditions, the upper boundary of the microchannel domain section is set with a impedance condition, to reflect the PDMS channel roof. All the vertical boundaries on the unit’s left and right edges are set to be periodic boundary conditions. The meshes on the two sides are also set to be identical.

According to Figure. S4, both coupling layer thickness and channel bottom thickness will affect the distribution of acoustic field in microchannel. The manipulation relies on the stable wave nodes to capture the micro targets, and high pressure gradient to provide acoustic gradient force. This calls for the highly efficient acoustic energy transfer and low distortion on acoustic field pattern. As shown in Figure. S4**b**, as the thickness of coupling layer increase from 50 μm to 250 μm, with 50 μm as a step size, the distortion of the pressure field in microchannel gradually increase. It is obvious that, when the deionized water coupling layer reach 250 μm, in the symmetric mode, the boundaries of the pressure regions become blurred in microchannel, and their pattern is no longer consistent with that of surface acoustic wave on the piezoelectric substrate. Similarly, as shown in Figure. S4**c**, as the thickness of channel bottom increase from 50 μm to 250 μm, with 50 μm as a step size, the pressure amplitude in microchannel gradually decrease. In Figure. S4**b** and **c**, the simulation results in channel bottom and coupling layer share the same legend. In each figure, the three legends, from top to bottom, are the pressure in microchannel, the pressure in coupling layer and channel bottom, and the displacement in piezoelectric substrate.

2. Cancer cells manipulation

In this study, we demonstrated a series of cancer cell manipulation experiments, including the translation, rotation, clustering, and selective collection. Cell rotation is one of the most important techniques for cell manipulation in modern bioscience, as it permits cell observation from any arbitrary angle and simplifies the procedures for analyzing and microsurgery ^[7]^. For cancer cell manipulation, in vertical plane, cells can be levitated to a stable position due to the balance of gravity, buoyant force, acoustic radiation force, and drag force from microstreaming ^[8]^. By moving the acoustic field, the torque generated by acoustic radiation force and drag forces can lead to cell cluster rotation (Figure. 4**d**). Simplifying a cell cluster with irregular structures to an ellipsoid with a long axis and a short axis, it is easier to rotate along its own long axis. After capturing the cell cluster in acoustic trap, moving the transducer along the cluster’s short axis can rotate the cell cluster.

For the dotted standing field generated by two orthogonal pairs of IDTs, the cells are trapped in isolated acoustic wells, and closely gathered into cell clusters due to the acoustic interaction force. For each cell among the dispersed cells in the standing wave field, its total acoustic pressure includes the external pressure field from the transducer, and the partial scattered pressure from the other cells. For the cell 1~N in the acoustic field, the total pressure $p_{t}$ of the cell $q$ is [9]:

$$\begin{aligned} p_{t}=p_{0}+\sum_{\begin{aligned} n=1 \\ n\neq q \end{aligned}}^{n=N} p_{n}\#\left( S7 \right) \end{aligned}$$

The acoustic interaction force determines how the cells interact with each other and the formation of the cluster, while the primary acoustic radiation force keep the cell cluster inside the acoustic traps (Figure. 4**e**). These cluster can be precisely translated through acoustic field movement, and regroup through acoustic field shaking (Figure. 4**e**). The evolution of acoustical agglomeration is under the action of radiation and hydrodynamic forces. When cell clusters move with the acoustic field, they are subjected to acoustic radiation force and drag forces in opposite directions, and thereby have a specific $v_{m}$ under this balance. With Stokes drag force linearly related to cell cluster size, the radiation force in a cubic relationship with the cell clusters sizes enables larger clusters to have higher $v_{m}$, which allow them to follow the movement of acoustic field, while the smaller clusters hindered by drag force. Therefore, by carrying large cell clusters to smaller cell clusters with an acoustic field at a higher speed, multiple cell clusters can aggregate into larger ones. In order to improve efficiency, in practical experiments, this process can be simplified into the high-speed shaking of the acoustic field to bring cell clusters at different positions closer and aggregate to each other.

In this study, to realize the precise manipulation of individual target (Figure. 6), we use the SAW tweezer to hold micro “tool” to indirectly manipulating micro targets. To do that, two problems need to be solved: firstly, how to make the tool and target have different motion trajectories in the same acoustic field, and secondly, how to enable tools to manipulate targets.

For the first problem, we utilize the difference in acoustic properties between air bubbles and cancer cells. In Eq. S2, the acoustophoretic contrast factor $\Phi$ is a coefficient determined by the relative density and relative compressibility of the fluid environment and the manipulated objects ^[2]^:

$$\begin{aligned} \Phi\left( \tilde{\kappa},\tilde{\rho} \right)=\frac{1}{3}\left[ \frac{5\tilde{\rho}-2}{2\tilde{\rho}+1}-\tilde{\kappa} \right]\#\left( S8 \right) \end{aligned}$$

where the compressibility $\kappa= 1/{\rho c^{2}}$. Therefore, objects with different sound speeds and densities can experience different acoustic radiation forces. In this case, the sound speed of air at room temperature is about 340m/s, with a density of 1.29 kg/m³, which leads to an acoustophoretic contrast factor $\Phi$ about -4674.297. As a comparison, human soft tissues have similar density to deionized water, and a sound velocity about 1540 m/s, which leads to an acoustophoretic contrast factor $\Phi$ about 0.039, significantly smaller than that of air. Hence, with the mismatch on radiation force, we can independently manipulate bubble with little influence on cells.

For the second problem, we utilize air bubble as a scatterer sound source for pressure waves, reflecting waves from IDT, to attract and collect cells with acoustic interaction force. The external driving frequency (18 MHz) is much higher than the bubble resonance frequency, so the radial responses of the bubble can be neglected. The high acoustophoretic contrast factor gives bubble a high sound energy reflection, allows it to generate high energy density acoustic field in the original SAW field. The acoustic interaction force $\boldsymbol{F}_{ai}$ is a function related to the distance $\boldsymbol{\varrho}$ between the sound source and the target. For a rigid particle target, the time-averaged radiation force $\boldsymbol{F}_{ai1}\left( \varrho\right)$ it got from an oscillating bubbles can be expressed as ^[10]^:

$$\begin{aligned} \boldsymbol{F}_{ai1}\left( \varrho\right)=\frac{4\pi\rho_{0}u_{0}^{2}r_{t}^{3}r_{s}^{4}\omega^{2}}{\varrho^{5}}\cdot\frac{\rho_{t}-\rho_{0}}{{2\rho}_{t}+\rho_{0}}\#\left( S9 \right) \end{aligned}$$

where the subscripts “s” and “t” here and in the following relates to the sound source object, and the target object, respectively. $\omega$ is the angular frequency. $u_{0}$ is the surface vibration velocity of the bubble. For a cancer cell, it has fluidity, elasticity and stable shape. When the bubble come near the cell to collect it, the distance $\varrho$ is in the short-range limit, that is, the dimensionless target-source distance parameter $k\varrho\ll1$. Assuming that the air bubble and its target are in a standing plane wave, and the target is in the same XY plane with the bubble sound source, the acoustic interaction force $\boldsymbol{F}_{ai2}$ can be expressed as follows ^[11]^:

$$\begin{aligned} \boldsymbol{F}_{ai2}\left( \varrho\right)\approx-\frac{4\pi E_{0}k^{2}r_{t}^{3}r_{s}^{3}\kappa_{t}\kappa_{s}}{9\kappa_{0}^{2}\varrho^{2}}{sin}^{2}(kh)\boldsymbol{e}_{\varrho} , k\varrho\ll1\#\left( S10 \right) \end{aligned}$$

$E_{0}$ is the characteristic energy density of the external standing plane wave, and $E_{0}=(1/2)\rho_{0}v_{0}^{2}$, where $v_{0}$ is the magnitude of the oscillatory velocity. $h$ is the distance from the first wave node to the origin of the coordinate system. The Eq. S10 can roughly describe the acoustic interaction forces generated by the bubbles. As the bubbles used in this study are greater than the Rayleigh scattering limit, and the target is a cell instead of an idealized fluid spherical target, the actual situation in practice is more complex.

In fact, for the second problem, “how to enable tools to manipulate targets”, we can use any microscale object as a tool, as long as it has significant acoustic impedance differences. Even without considering acoustic interaction force, these objects can still be used to push cells to specific positions, in which way, the virtual acoustic tweezers are like holding a physical “shovel” to push cells around. This approach is very simple and feasible, and can be derived into various forms: by replacing the micro tool with another structure. However, using bubble as a tool has its special effects. Bubbles not only generate acoustic interaction force to actively attract cells, providing many conveniences for cell collection, but also generate intense scattered fields, fundamentally changing the local acoustic field distribution.

As the source sphere, the air bubble can exhibit a strong monopole scattering, and a new patterned acoustic field can be formed by the coupling of the scattered field from bubbles and the plane standing waves. The scattered field has a geometric distribution of concentric circles centered on the bubbles, with acoustic energy gradually decreases from the inner circle to the outer circle ^[10]^. To visually display the distribution of the coupled acoustic field, related simulation and experiments have been carried out. As shown in Figure. S5, the bubble inside channel can locally alter the acoustic field distribution. We have established a model with an air bubble in the center section of the microchannel to analyze the scattered field (Figure. S5**b**). This model only analyzes acoustic pressure and does not involve the acoustic streaming generated by bubble vibration. Two kinds of bubble are used in this model, one is a 70 μm bubble (Figure. S5**c**), consistent with the subsequent cell experiments in Figure. S5**e**; another is a 175 μm bubble (Figure. S5**d**), consistent with the bubble manipulation experiments in Figure. 5. Under the influence of the scattered field, the originally dotted wave field becomes circular, with arc-shaped high-pressure and low-pressure region centered on the bubble (Figure. S5**d**). In the cell collecting experiment, the aggregation position of the cells can roughly mark out the approximate position of the wave node. Under an input power of about 7.2 W, the coupled fields can construct patterned wave nodes around bubble (Figure. S5**e**). In Figure. S5**e**, all black concentric circles have the same spacing of 50 μm, a quarter wavelength. The black concentric circles do not indicate the actual wave node positions, but serve as auxiliary lines to facilitate the observation of the relative positions among cell clusters and the bubble. The four intersecting dashed lines are spaced 45 ° apart, and the intersection point coincides with the center of the bubble. The Figure. S5**f** and **g** are close-up images of Figure. S5**c** and **d**, respectively. It is magnified to the same scale as Figure. S5**e**. In Figure. S5**e** to **g**, these three sets of concentric circles are exactly the same, and both centered around bubbles. By comparing the Figure. S5**e** and **f**, it can be observed that the location of cell aggregation in Figure. S5**e** corresponds to the wave node in Figure. S5**f**. The cells no longer aggregate into a dot-shape cluster like that in dotted SAW field, but form a cell line, with its perpendicular bisector roughly passing through the center of the bubble. This confirms that the wave nodes of the coupled field form a symmetrical circular patterned distribution around the bubble. For coupled acoustic fields, the newly constructed wave nodes have different pressure gradients, which also means that the cells within them have corresponding $v_{mtt}$ and $v_{m}$. Under the manipulation of the SAW field, when the bubbles move beyond this speed, they can catch up with the cells and collect them. On the contrary, when bubbles move slowly, slower than $v_{m}$, and gradually change the pressure distribution with scattered waves, the cells can move with the potential wells of the coupled field. For acoustic waves in microchannel and on piezoelectric substrate surface, their wavelengths are $\lambda_{a}$ and $\lambda_{s}$, respectively, and $\lambda_{a}<\lambda_{s}$. For the plane standing field in the channel, its pattern in XY plane follows the distribution of surface acoustic waves on piezoelectric substrate surface, and has a spatial period of $\lambda_{s}$ along X or Y direction, even though its wavelength is $\lambda_{a}$. For the scattered wave around the bubble, it propagates in the wavelength of $\lambda_{a}$, forming a field pattern follows the contour of the bubble. In the XY plane, the short attenuation length of reflected wave leads to a rapid dissipation of sound energy, and the scattered field does not change the entire acoustic field, but rather the local area around the bubble. Besides, the interaction force is an attractive force inversely proportional to the square of the distance between sound source and target, so the bubble is only effective to collect the nearby cells. Through this coupling method, movable SAW tweezer can dynamically control the acoustic field distribution in different regions, reconstruct cell clusters layout patterns, and freely decide which cells to be collected by moving the bubble near them.

For the observed cell rotation, as shown in the experiment of Figure. 5**d**, the cells and bubble may rotate in the acoustic field when the bubble size is small. Before cells are collected, the bubble sphere is located initially in the center of the wave node, resulting in zero primary force. When cells are adsorbed on the bubble surface by interaction force, this “bubble-cells” system will result in a collective rotation with the direction depending on the system shape. When translate the bubble in the fluid, the drag force causes cells to move along the outer contour of the bubble, which change the system shape and the rotation direction ^[9]^.

In this study, bubbles are only used as an example to demonstrate the method that relies on acoustic interactions to collect cells and uses scattered fields to alter cell patterns. Using other microstructures, such as spiral microstructures, as tools, can achieve different effects, which can aggregate cells into clusters of different shapes and generate unique coupled acoustic fields within the SAW field to achieve dynamic reconfiguration of cell layout patterns.

3. Manipulation of particle in complex microchannel environment

In order to demonstrate the versatility and practicality of this movable acoustic tweezer, we further demonstrated its manipulation performance on particles of different size, as well as its performance in complex channel structures.

The SAW field has a selective effect on particles of different sizes: on the one hand, the acoustic radiation force increases exponentially with particle size; on the other hand, the particles much smaller than the wavelength can pass through the barrier composed of acoustic pressure along wave nodes in the standing field, without being blocked by the anti-nodes. For example, in a standing field, for a particle has a diameter greater than 0.15$\lambda_{s}$, the force along the nodal line can act to repel the particle from passing through ^[12]^. In the experiments shown in Figure. 1, the particle is 31.1 μm, merely larger than 0.15$\lambda_{s}$ ($\lambda_{s}$ = 200 μm). Here in Figure. S6**a**, we demonstrated the manipulation of particle with a diameter of 10 μm, about one-third of the size of the particle in Figure.1 and only 0.05$\lambda_{s}$, which causes it to experience far lower acoustic radiation forces. The 10 μm particle can be moved along specific path to depict letters, at an input power of 7.2 W. It shows that, although the movable SAW tweezer choose a rather low frequency and long wavelength ($\lambda_{s}$ = 200 μm, obviously larger than most of cell samples) to reduce sound energy dissipation and wavefield distortion in the multilayer structure, its applicability on sample size can still cover a large range, from 10 μm (one twentieth of $\lambda_{s}$, close to most of human cells’ size) to more than 100 μm (for example, a cell cluster composed of dozens of cells). The pattern formed by particle trajectories is made of screenshots of particle motion videos. In order to prevent the moving transducer from obstructing the field of view or covering the particle trajectory image, the background of the final figure is selected from the image when the transducer’s electrodes are not in the center of field of view. The auxiliary lines and image in the particle trajectory figure, including the “XIAN” in Figure. S6, the “SAW” in Figure. 4 and the Chinese characters in the following Figures of spirulina manipulation experiment, are all digital shapes added in the figure after experiments, and do not exist in the microchannel in the experiments.

In order to achieve excellent manipulation performance, most experiments are conducted in a circular chamber with a diameter of 8 mm (Figure. 1~5), which are far larger than the wavelength and the acoustic window, to ensure the resonance of the acoustic field and the stable distribution of acoustic pressure. Compared to large chambers, the narrow channels make it more difficult to manipulate particles, as the acoustic field orientation inside can be effectively changed via microchannel diffractive effects [13]. Traditionally, manipulating particles’ motion in narrow microchannels typically requires a long action distance [14] and the particles gradually change their motion direction under the continuous action of multiple nodal lines. However, for movable SAW tweezer, in order to achieve high-precision and controllable manipulation, it is necessary to keep particles moving closely with the potential well, which means a far shorter action distance. In the experiment in Figure. S6**b**, we move a 31.1 μm particle freely in microchannels, and put it into a branch channel about 100 μm wide, which is about half the $\lambda_{s}$. The PDMS channel wall and channel bottom can simulate blood vessels with different sizes and shapes, proving the device’s potential to be used in micro targets control within animal vasculature ^[15]^. Furthermore, we manipulate a 15 μm particle inside channel with complex structure, including cross channel and curved channel (Figure. S6**c**). Compared to straight channel, the diffractive effects on the curved channel wall will disrupt the distribution of wave nodes to a greater extent, modulating the pressure field based on the channel’s geometry ^[15]^. The channel in Figure. S6**c** used the borosilicate glass sheet channel bottom, different from the channel setup in Figure. S6**b**. Although the PDMS bottom can have better acoustic waves propagation and is suitable for packaging narrow channels, the sound velocity difference between the glass bottom and the fluid can simulate the influence of interference factors such as bones.

4. Manipulation of biotargets with helix structures

Apart from spherical targets experiments shown above, helical targets are also put into tests to demonstrate the versatility of the movable acoustic tweezers (Figure. S7**a**). Spirulina is a kind of spiral-shaped multicellular micro alga. Its helix pitch varies from 12 to 72 μm, and helix diameter varies from about 30 to 70 μm [16]. One spirulina is composed of multiple spirals, and its total length can reach hundreds of micrometers, which is close to or bigger than the size of a wave node. As shown in Figure. S7**b**, spirulina with different lengths and different helix diameter can all be precisely moved and form specific patterns. Furthermore, we control the spirulina to depict a very complex pattern that composed of four Chinese characters: “声表面波”, which means surface acoustic wave (Figure. S7**c**). The entire process took 301 seconds, and the device did not overheat, lose efficacy or lose control of the target. Due to nonlinear coupling between thermal and acoustical mode, SAW can induce quick temperature increase in microchannel when it’s bonded to the piezoelectric substrate [17]. In this system, the extra coupling layer helped to reduce the heat transmission from the piezoelectric substrate and avoid temperature rise in channel.

Compared to particles, spirulina has a larger size and therefore cannot be captured within a single wave node, but rather within a wave node line surrounded by multiple antinodes (Figure. S7). When the acoustic field rotates, the imbalance of the acoustic radiation forces at both ends of the spirulina makes it to follow the pressure nodes’ motion and generate in-plane (XY plane) rotation. The movement of manipulated samples are far slower than the acoustic field changing in MHz, whose low-pressure area and high-pressure area switch position every half a cycle. Thus, besides the transient distribution of acoustic pressure field, the absolute value distribution ^[12]^ and the time average value distribution of acoustic pressure can be used to display the position of wave nodes and can intuitively describe the geometric distribution of a standing field. The schematic diagram of pressure field in Figure. S7**d** is the absolute value distribution of the time-averaged acoustic pressure value of the 20th cycle. Considering the computing efficiency, all transient simulations are calculated till the end of the 20th cycle, whose time consumption is 1.5 to 8 hours on a high-performance workstation, varies with calculation content and model. The microstructure on the spiral targets can determine a length scale much smaller than the whole target itself, which can lead to a more complex effect from acoustic radiation force and acoustic streaming ^[18]^. The precise translation and in-plane rotation of a micro spiral structure can prove the practicality of this device, and its potential to drive microrobot with special shapes ^[18]^. The input power in the experiments of Figure. S7 is 5.0 W.

5. Manipulation of large samples in an open environment

All the manipulation experiments above were conducted in closed microchannels, which can provide stable microfluidic environment and maintain laminar flow distribution, but the microchannel also limits the size of the manipulated sample. A roofless channel, or just the channel bottom, can provide an open environment for larger targets manipulation. As shown in Figure. S8**a** and **b**, the sessile droplets can be transported by travelling waves, and fused by standing waves. The sessile droplets are approximately 2 mm in diameter, about ten times the wavelength $\lambda_{s}$. The droplet in Figure. S8**a** is directly put on the glass sheet. The droplets’ motion is due to the contact angle difference between the front and rear interfaces, and thus to the asymmetry of the drop, and its velocity shall be proportional to the surface acoustic wave power [^19^]. The droplets’ vortex inside is due to the acoustic streaming driven by the bulk wave radiating in the liquid as the acoustic waves passes underneath the droplet [20]. The bulk wave gradually transfers its pseudo-momentum to the fluid as it is attenuated.  By only turning on one transducer of the paired IDTs, droplets can be pushed along the direction of the travelling acoustic waves, and by moving and rotating the transducer, the droplets can be transported to further places in any direction. In Figure. S8**a**, the droplet is gradually pushed away bit by bit, with an input power of 1.9 W. In Figure. S8**b**, the glass sheet is covered with fluorocarbon oil (FC-40) film, and upon which placed the droplets. On the one hand, the acoustic attenuation of locally coupled waves generates acoustic streaming inside the oil film that can affect droplets’ motion[21]. On the other hand, when subjected to the acoustic waves around 20 MHz, the millimeter sized droplets will reflect and scatter the wave field, producing oscillations that is about 10 to 200 Hz [22]. The acoustic interaction force between the two droplets causes them to approach each other and eventually fuse ^[9]^.

Precise rotational manipulation of microscale organisms is an essential capacity in biotechnology, and is of great significance for the multi angle observation and 3D modeling of cells, multicellular organisms, microtissues and microorganisms ^[23]^. Although researchers have developed numerous micro manipulation techniques, performing controllable rotational manipulation has remained a significant challenge ^[24]^. Zebrafish exhibit significant physiological and genetic parallels to humans, serve as high-throughput and cost-effective alternatives models in drug screening processes, and its rotational manipulation is a crucial capability to extract valuable anatomical information and achieve multidimensional analysis, regarding which issue, Zhiyuan Zhang et al. provided a series of practical and excellent body waves methods ^[25]^. In this study, we attempt to performed controllable rotation operations on zebrafish eggs with surface wave method. For the zebrafish eggs (Figure. S8) which are much larger than the wavelength, traveling waves can be used to rotate them in XZ plane, with an input power of 0.13 W. For the travelling field in the experiment of Figure. S8, the radiation force’s scattering component dominant over its gradient component when pushing the fish egg along the direction of the SAW. The acoustic scattering forces $F_{s}$ is ^[4]^:

$$\begin{aligned} F_{s}=\frac{nP}{c_{o}}\left( 1+R\cos(2\theta_{i})-\frac{T^{2}\left( cos\left( 2\theta_{i}-2\theta_{r} \right)+Rcos\left( 2\theta_{i} \right) \right)}{1+R^{2}+2Rcos\left( 2\theta_{r} \right)} \right)\#\left( S11 \right) \end{aligned}$$

The torque formed by acoustic scattering force and the friction force on the glass sheet causes the fish egg to rotate. Besides, traveling waves generate acoustic streaming in the water film on the glass sheet. Considering that the size of the zebrafish egg is about 6 times the wavelength $\lambda_{s}$, sound waves will experience rapid attenuation within this 1.2 mm distance, and the acoustic streaming will produce different distributions on the two sides of the fish egg. On the side near the electrodes, the acoustic streaming in the same direction as the travelling acoustic beam will push the fish egg with drag force, while on the side away from the electrode, the acoustic streaming is less intense, due to the acoustic attenuation and obstruction by the fish egg. In addition to one-step rotation (Figure. S8), the zebrafish eggs can be gradually and precisely rotated. As shown in supplementary videos, by constantly opening and closing the acoustic field, and controlling the position of the transducer, the fish egg can generate out of plane rotation (XZ plane) bit by bit, and display its inner structure from different angles. Boundaryless rolling manipulation is fairly important for living organisms and engineered systems at microscale, which can break the spatial homogeneity of surrounding mediums ^[26]^. In the experiment, the required input power to drive zebrafish eggs was relatively small compared to other experiments in this study. Under high input power and radiation force, zebrafish eggs can be easily pushed away along the acoustic beam. Controlling input power allows us to control whether to translate or rotate the fish egg. By combining translation and rotation operations, zebrafish eggs can be controlled to do complex movements without spatial limit.

Supplementary Figure


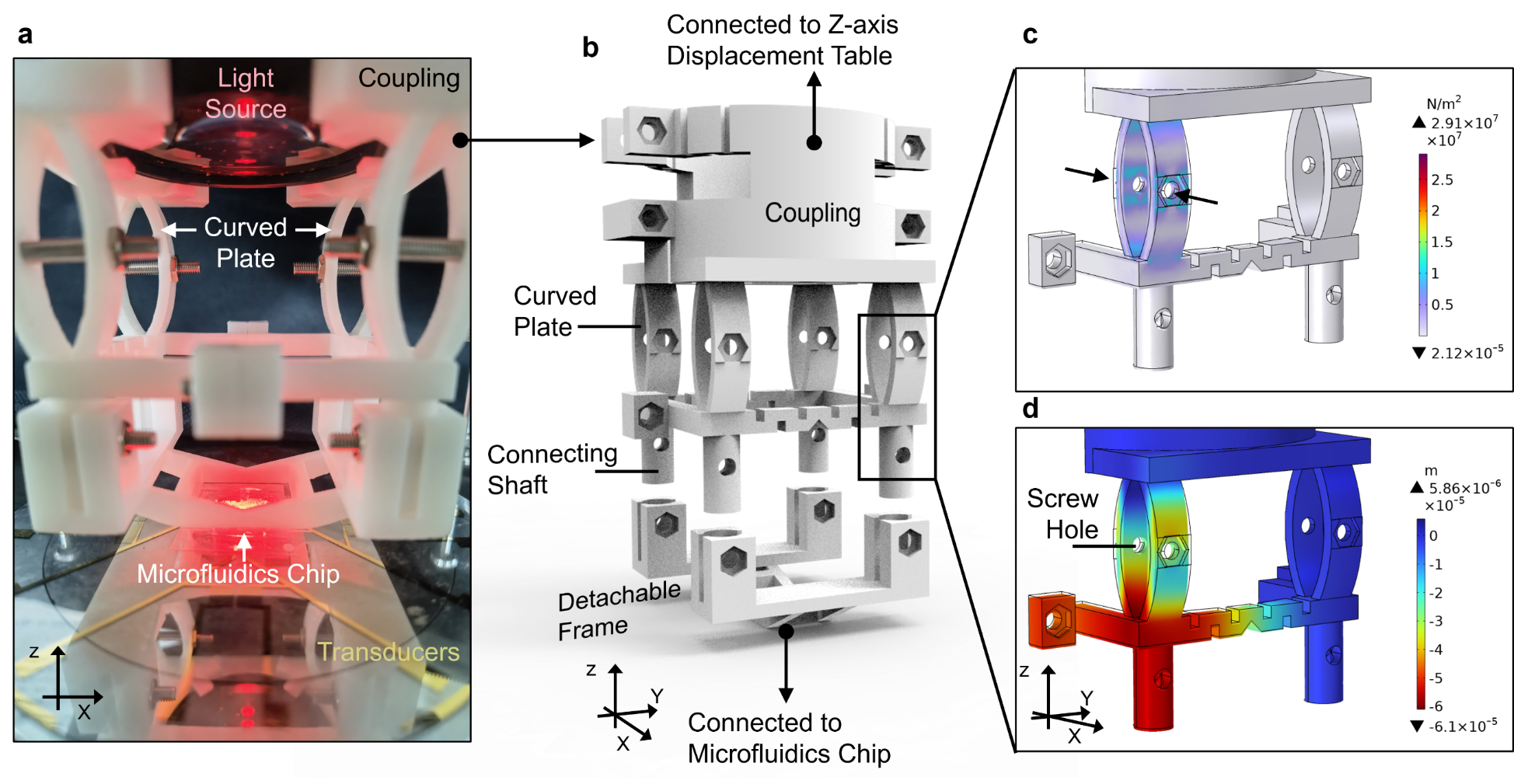


Figure S1: Schematic diagram of Clamp structure and method of height adjustment. a. Photo of movable acoustic tweezers system. b. Clamp structure. c. Simulation result of stress distribution when a displacement of 100 micrometers is applied at the screw hole. d. Simulation result of displacement along the Z-axis.


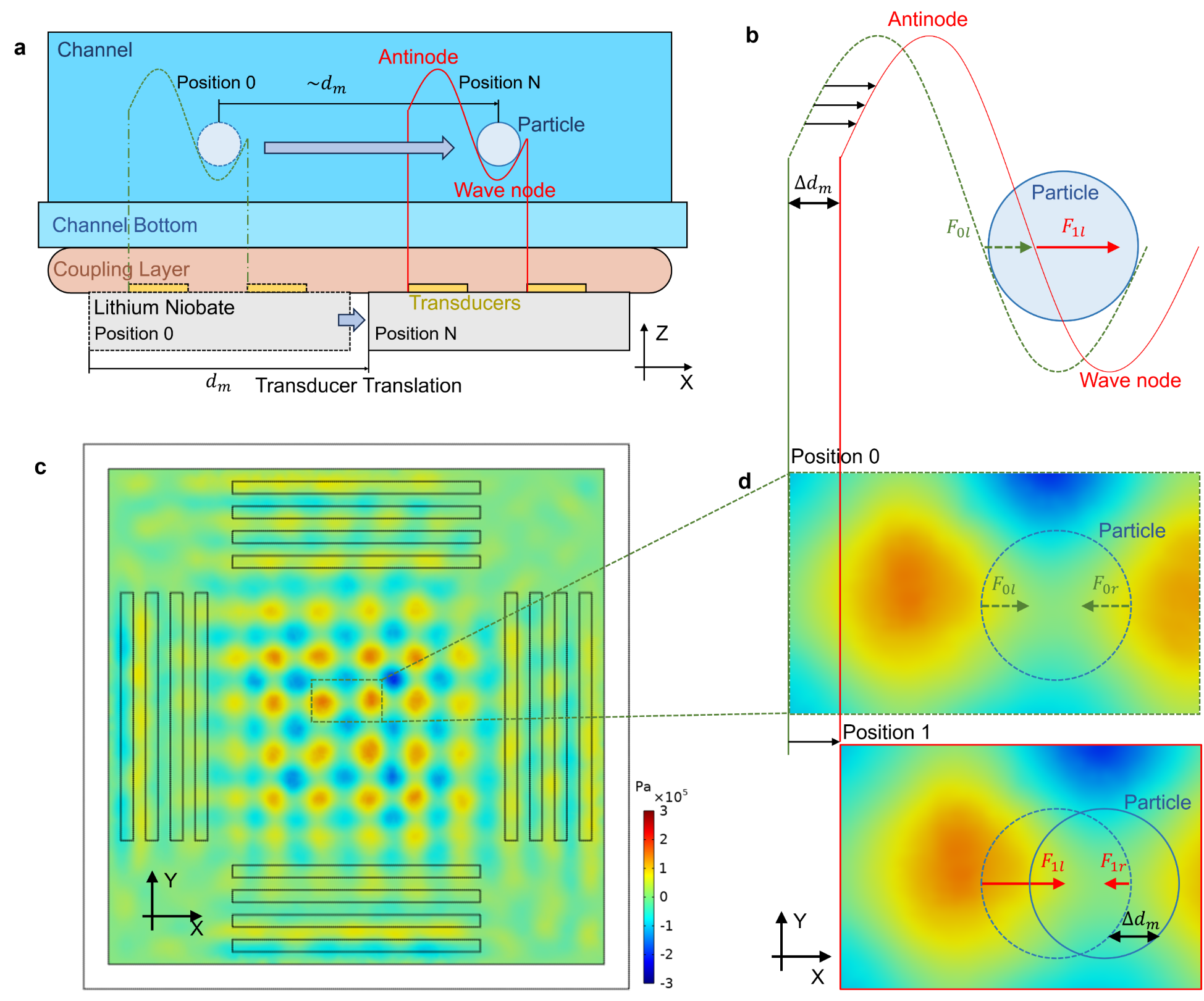


Figure S2: Particle translation method. a. The particle motion under mobile acoustic field. b. Schematic diagram of wave node movement. c. Simulation result of acoustic pressure in microchannel on XY plane. d. The imbalance of acoustic radiation force caused by the movement of wave nodes.


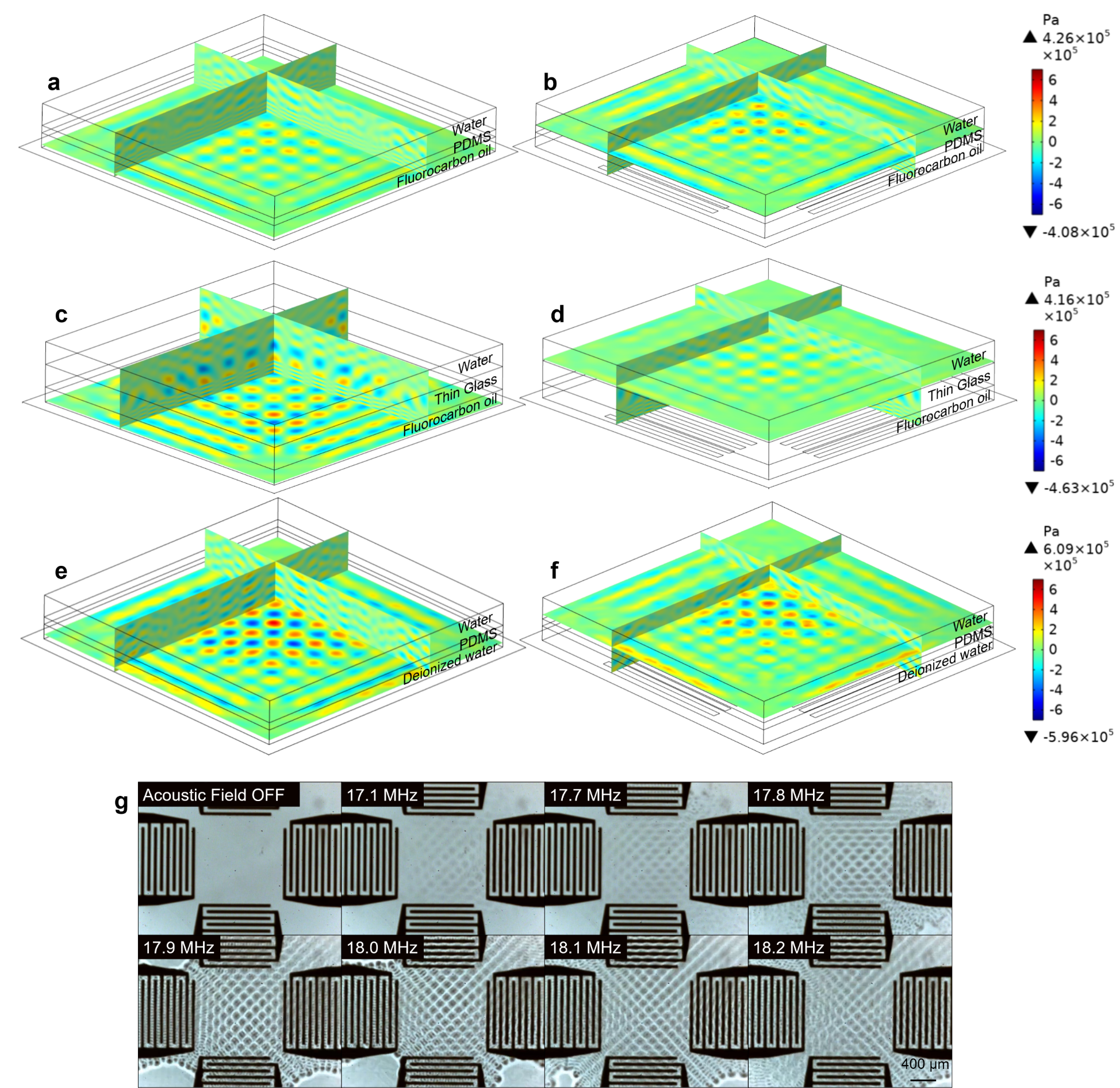


Figure S3: Acoustic pressure field distribution with different setup. The figures from a to f are the COMSOL simulation results. These 6 figures show the acoustic field distribution when the chip setup is different to that in Figure. 2. a. and b. The coupling layer is deionized water, and the channel bottom is glass sheet. c. and d. The coupling layer is fluorocarbon oil, and the channel bottom is glass sheet. e. and f. The coupling layer is deionized water, and the channel bottom is PDMS. a, c and e display the pressure field within the coupling layer and the changes in the spatial period of acoustic pressure on the Z-axis. b, d and f display the pressure field within the microchannel. g. The wave field generated by four IDTs at different frequencies. The pattern in the figure is the wavy surface of fluorocarbon oil film, excited by the leaky Rayleigh waves.


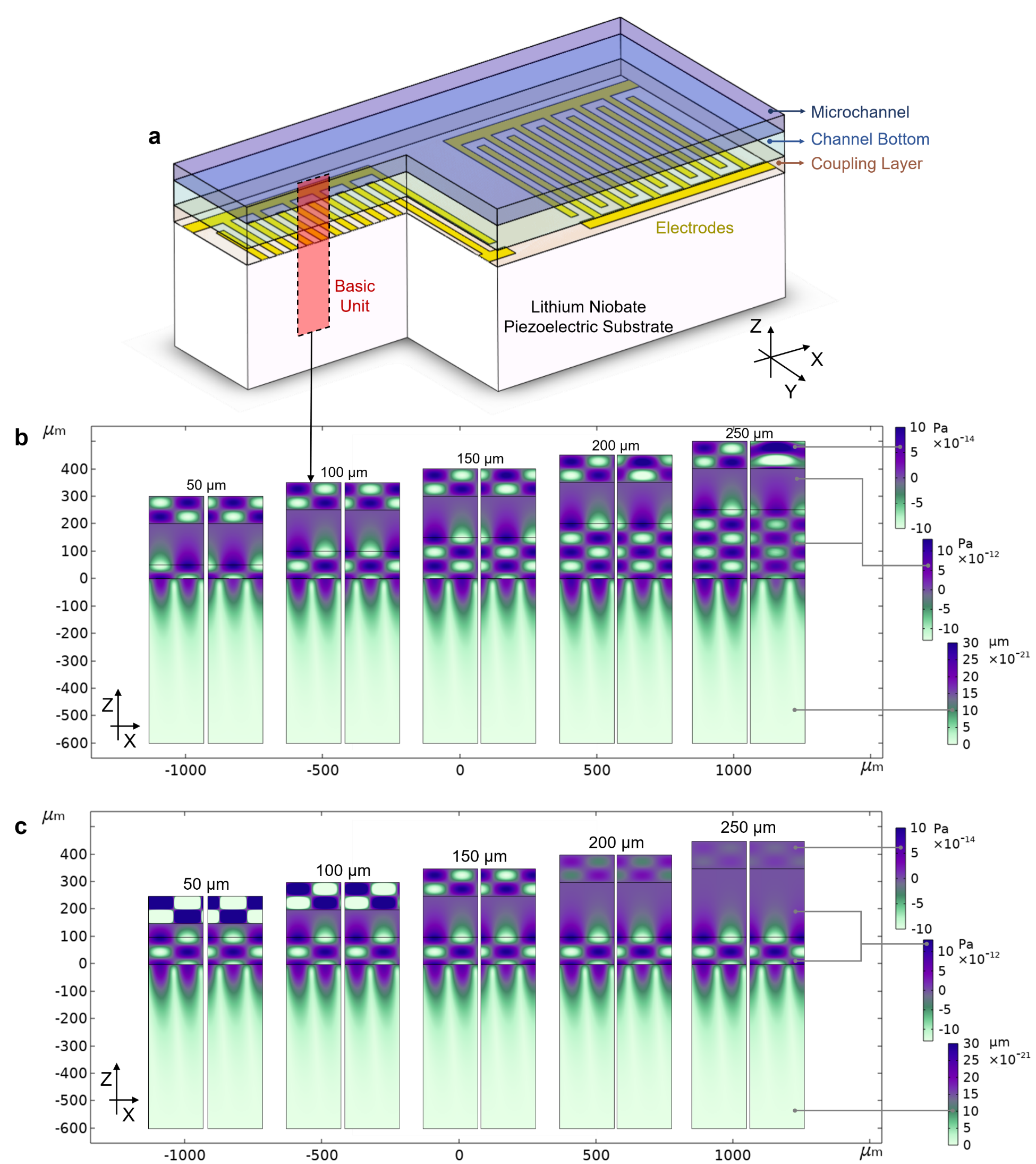


Figure S4: Simplified COMSOL simulation results of the multilayer structure. a. The simulated area is not the entire transducer, but a basic unit whose width determined by the wavelength and the spatial period of the electrode fingers. The distribution of the 4 layers of every figure in b and c, from top to bottom, are acoustic pressure of deionized water in microchannel, acoustic pressure of glass sheet channel bottom, acoustic pressure of deionized water coupling layer and displacement of lithium niobate piezoelectric substrate, respectively. The channel bottom and coupling layer layers share the same legend. All figures in b and c are arranged in pairs, and for the two figures in every pair are the two SAW modes, antisymmetric and symmetric. The figure b and c show the influence of the thickness of coupling layer and channel bottom, respectively. In Figure. S4b and c the size number, 50 μm to 250 μm, refer to the thickness of the coupling layer and channel bottom, respectively.


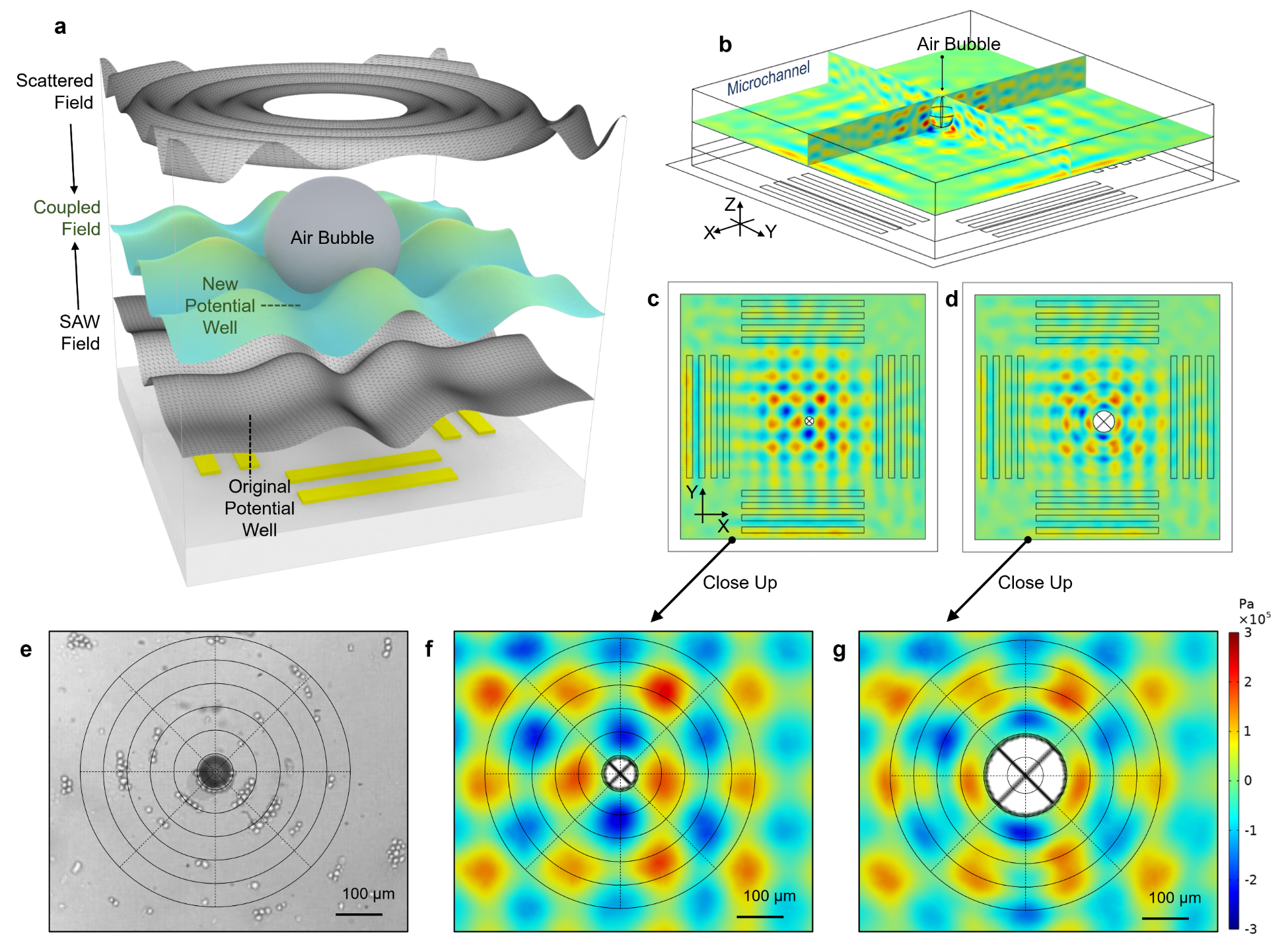


Figure S5: The coupling of the scattered field from bubbles and the plane standing waves. a. The schematic diagram of acoustic fields coupling. b. In the simulation, the bubble is placed inside the microchannel, and the geometric center of the bubble coincides with the middle section (XY plane) of the microchannel. c. The acoustic pressure result with a bubble of 75 μm. d. The acoustic pressure result with a bubble of 175 μm. The sound waves reflected by the bubble alter the acoustic field distribution in XY plane. e. In the experiment, cells captured and arranged by the bubble. The black lines in the figure are concentric circles with equal spacing. f. A close-up of the acoustic pressure result in c. g. A close-up of the acoustic pressure result in d. All the simulation figures in Figure. S6 share the same legend, the one on the right of figure g.


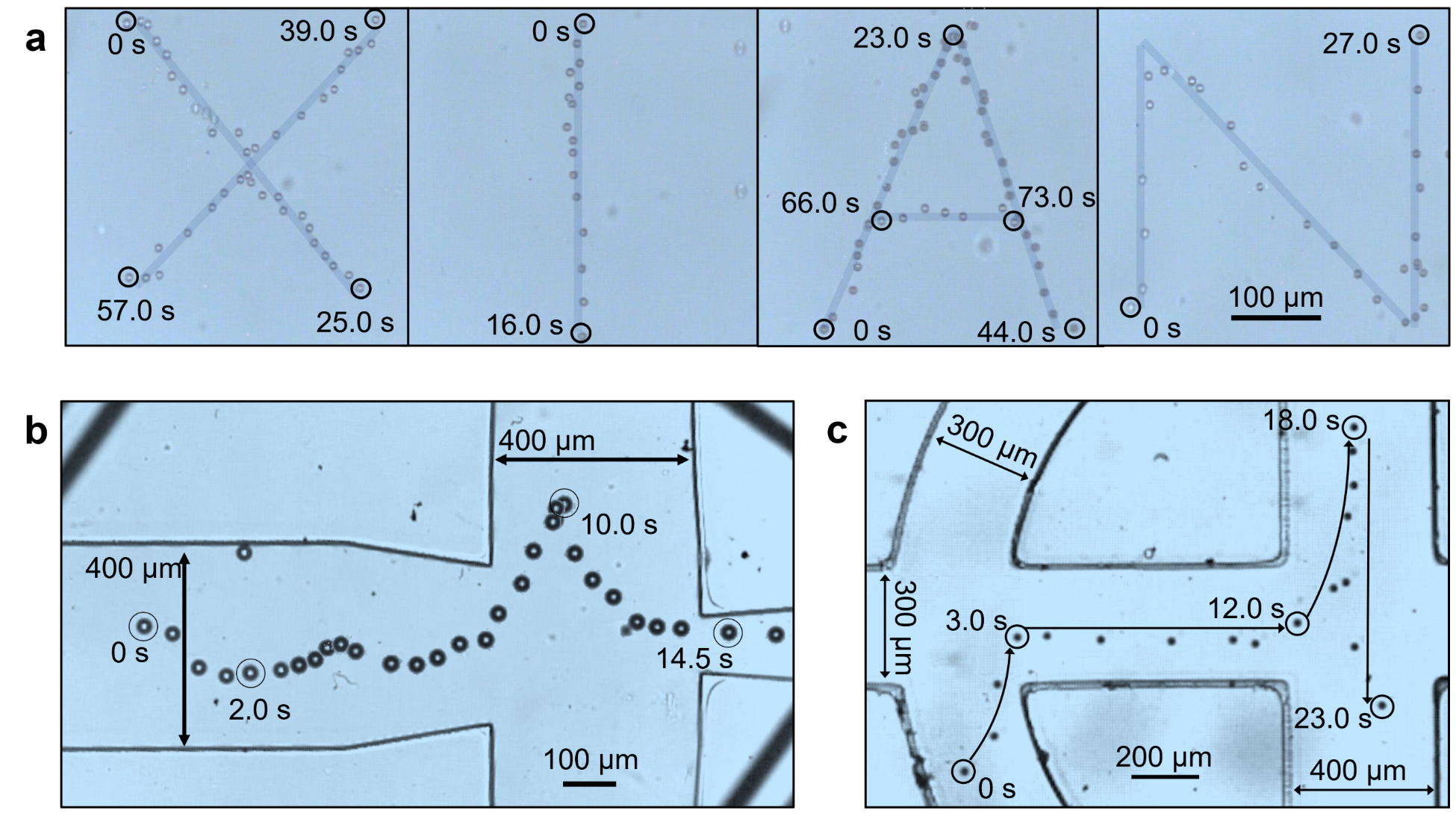


Figure S6: Arbitrary controlled motion of particles. a. Using particles to draw 'XIAN', which is the name of the city where both Xidian University and Xi'an Jiaotong University locate, and the middle name of one of the authors. b. Place a particle into a narrow channel. The channel setup in b is PDMS channel wall with PDMS channel bottom, while all the other channel in this study used borosilicate glass sheet channel bottom. c. Particle translation in microchannel with complex structure.


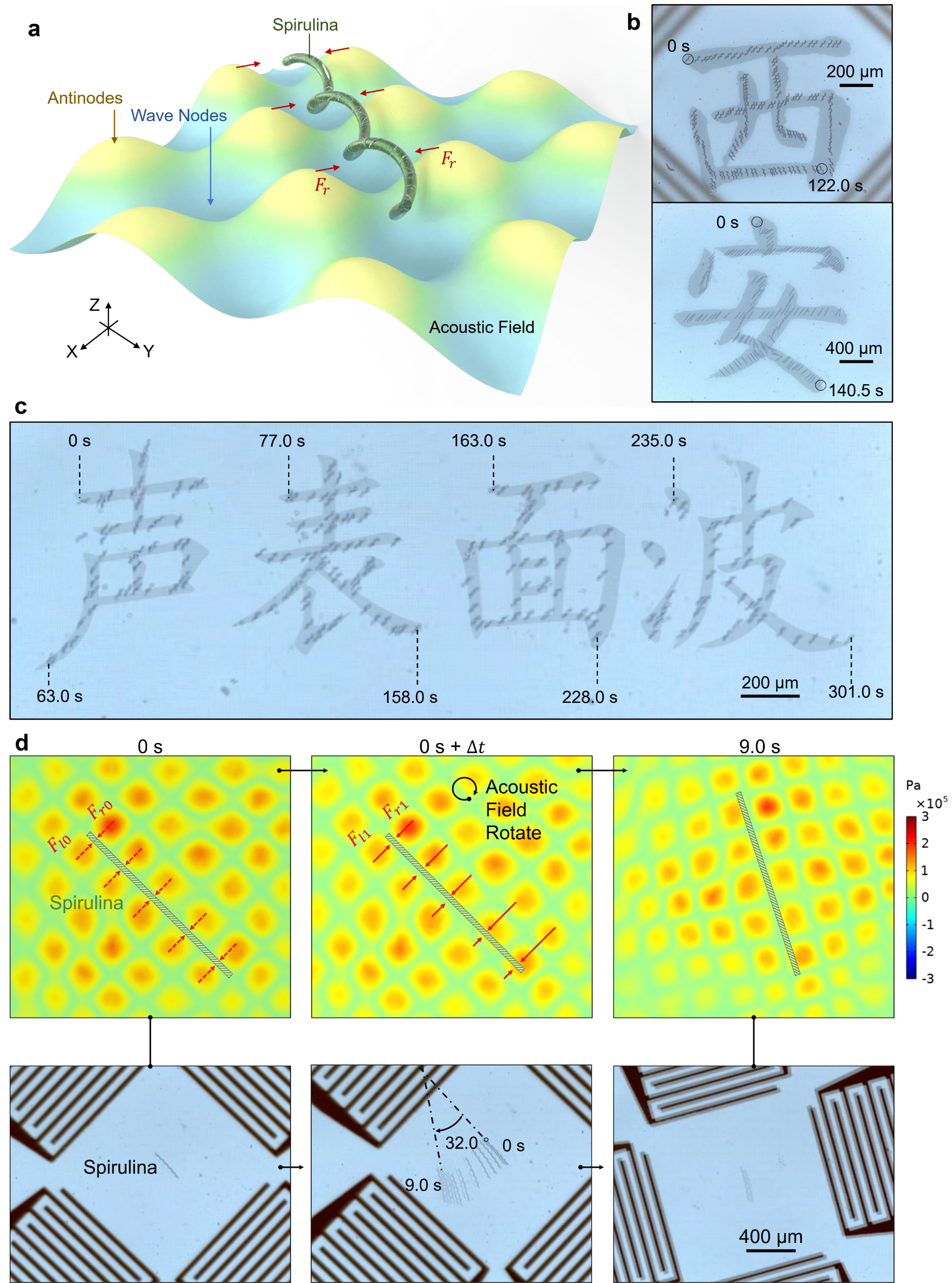


Figure S7: Spirulina manipulation. a. Schematic diagram of spirulina manipulation in acoustic field. b. Manipulating spirulina of different sizes. The two characters are Chinese language of 'Xian'. c. Continuously manipulating spirulina for more than 5 minutes to depict complex Chinese characters. d. Rotating spirulina in XY plane.


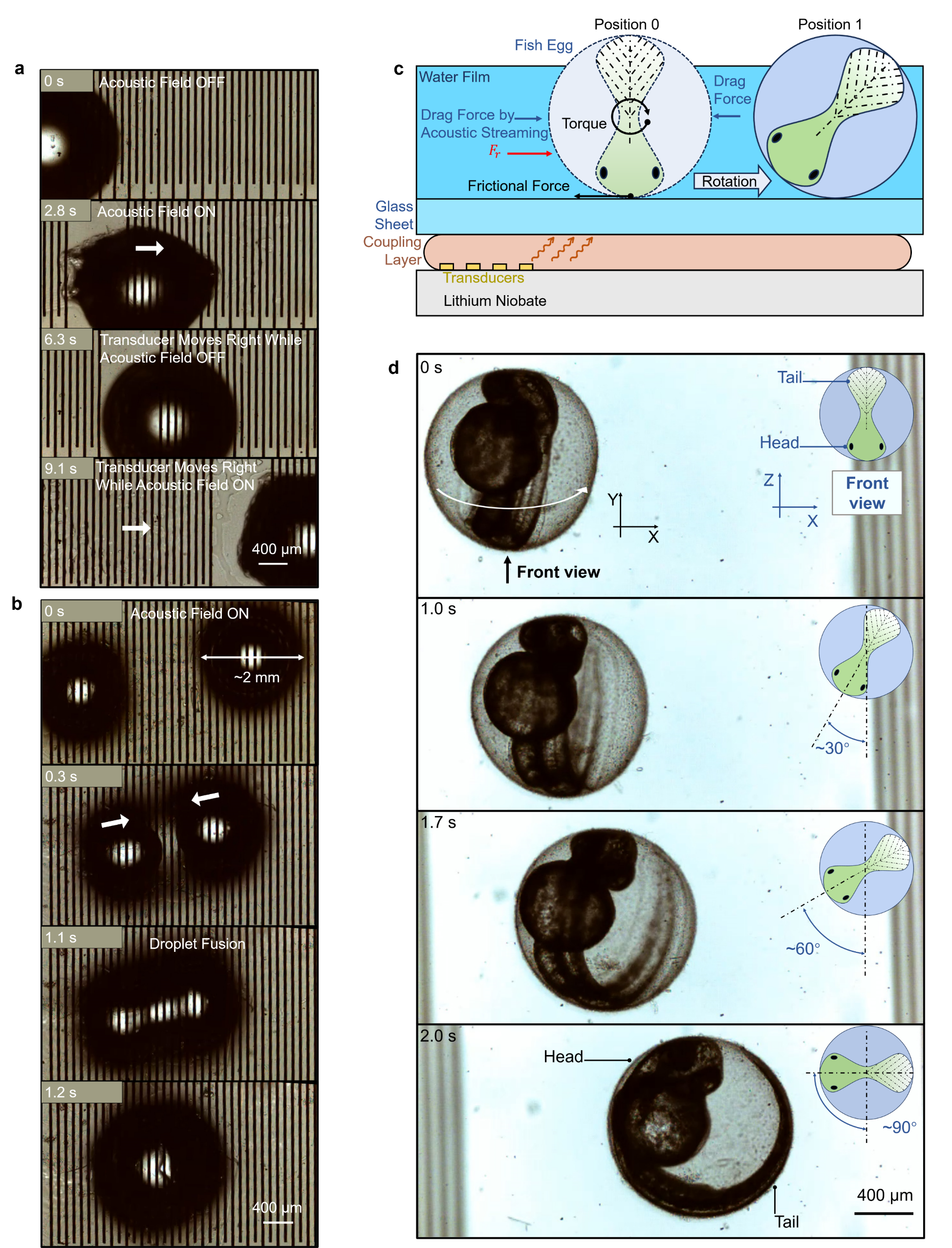


Figure S8: Manipulation of droplets and fish eggs. a. Sessile droplet pushing. b. Droplets fusion. c. Schematic diagram of fish eggs rotating in the XZ plane under the action of traveling waves. d. Rotation of a zebrafish egg in XZ plane.

Supplementary References

1. Dual, J., Hahn, P., Leibacher, I., Möller, D., Schwarz, T., & Wang, J. *Lab on a Chip* **2012**, 12(20), 4010-4021.
2. Bruus, H. *Lab on a Chip* **2012**, 12(9), 1578-1586.
3. Bruus, H. *Theoretical microfluidics (Vol. 18)* Oxford University Press, Oxford, 2008.
4. Lee, J., Ha, K., & Shung, K. K. *The Journal of the Acoustical Society of America* **2005**, 117(5), 3273-3280.
5. Mynard, J. P., Kondiboyina, A., Kowalski, R., Cheung, M. M., & Smolich, J. J. *Frontiers in Physiology* **2020**, 11, 1085.
6. Moustafa, M., Laouini, G., ElNaggar, M., & AlZoubi, T. *Ferroelectrics* **2021**, 572(1), 94-105.
7. Tang, T., Hosokawa, Y., Hayakawa, T., Tanaka, Y., Li, W., Li, M., & Yalikun, Y. *Engineering* **2022**, 10, 110-126.
8. Chen, K., Wu, M., Guo, F., Li, P., Chan, C. Y., Mao, Z., Li, S., Ren, L., Zhang, R., & Huang, T. J. *Lab on a Chip* **2016**, 16(14), 2636-2643.
9. Sepehrirahnama, S., Mohapatra, A. R., Oberst, S., Chiang, Y. K., Powell, D. A., & Lim, K. M. *Lab on a Chip* **2022**, 22(18), 3290-3313.
10. Miller, D. L. *The Journal of the Acoustical Society of America* **1988**, 84(4), 1378-1387.
11. Silva, G. T., & Bruus, H. *Physical Review E* **2014**, 90(6), 063007.
12. Fakhfouri, A., Devendran, C., Collins, D. J., Ai, Y., & Neild, A. *Lab on a Chip* **2016**, 16(18), 3515-3523.
13. Xu, M., Lee, P. V., & Collins, D. J. *Lab on a Chip* **2022**, 22(1), 90-99.
14. Peng, T., Zhou, M., Yuan, S., Fan, C., & Jiang, B. *Applied Mathematical Modelling* **2022**, 101, 517-532.
15. Del Campo Fonseca, A., Glück, C., Droux, J., Ferry, Y., Frei, C., Wegener, S., Bruno, W., Mohamad, E. A., & Ahmed, D. *Nature* *Communications* **2023**, 14(1), 5889.
16. Ali, S. K., & Saleh, A. M. Spirulina-an overview. *International journal of Pharmacy and Pharmaceutical sciences* **2012**, 4(3), 9-15.
17. Kondoh, J., Shimizu, N., Matsui, Y., Sugimoto, M., & Shiokawa, S. *Sensors and Actuators A: Physical* **2009**, 149(2), 292-297.
18. Deng, Y., Paskert, A., Zhang, Z., Wittkowski, R., & Ahmed, D. *Science Advances* **2023**, 9(38), eadh5260.
19. Baudoin, M., Brunet, P., Bou Matar, O., & Herth, E. *Applied Physics Letters* **2012**, 100(15).
20. Song, S., Zhou, J., & Riaud, A. *Physics of Fluids* **2022**, 34(8).
21. Zhang, P., Chen, C., Su, X., Mai, J., Gu, Y., Tian, Z., Zhu, H., Zhong, Z., Fu, H., Yang, S., Krishnendu, C., & Huang, T. J. *Science Advances* **2020**, 6(24), eaba0606.
22. Brunet, P., Baudoin, M., Matar, O. B., & Zoueshtiagh, F. *Physical Review E* **2010**, 81(3), 036315.
23. Läubli, N. F., Burri, J. T., Marquard, J., Vogler, H., Mosca, G., Vertti-Quintero, N., Naveen S., Andrew d. M., Ueli G., Daniel A. & Nelson, B. J. *Nature Communications* **2021**, 12(1), 2583.
24. Ahmed, D., Ozcelik, A., Bojanala, N., Nama, N., Upadhyay, A., Chen, Y., Wendy, H., & Huang, T. J. *Nature communications* **2016**, **7**(1), 11085.
25. Zhang, Z., Cao, Y., Caviglia, S., Agrawal, P., Neuhauss, S., & Ahmed, D. *Lab on a Chip* **2024**, 24(4).
26. Zhang, Z., Sukhov, A., Harting, J., Malgaretti, P., & Ahmed, D. *Nature Communications* **2022**, 13(1), 7347.
